# Bioactive chemical composition and pharmacological insights into *Salvia* species

**DOI:** 10.3389/fmolb.2025.1678109

**Published:** 2025-12-10

**Authors:** Tayyiba Afzal, Jarosław Proćków, Jacek Łyczko

**Affiliations:** 1 Department of Plant Biology, Institute of Biology, Wrocław University of Environmental and Life Sciences, Wrocław, Poland; 2 Department of Food Chemistry and Biocatalysis, Wrocław University of Environmental and Life Sciences, Wrocław, Poland

**Keywords:** medicinal plants, Salvia, plant extracts, secondary metabolites, essentialoils, salvianolic acid

## Abstract

Salvia is a genus of Lamiaceae family with more than 1,000 species having diverse utility. The wide range of uses encompasses food, flavor, cosmetics, aromatherapy, horticulture, and medicine. It has been attributed to the presence of bioactive compounds belonging to essential oils, phenolic compounds, and flavonoids that are extensively studied using spectroscopic and chromatographic techniques. This review aims to investigate in-depth previously published literature from 2020 to 2025 on 59 *Salvia* species. It was performed with several key search words focused on the chemical compounds in *Salvia* spp. and their pharmacological efficacy. *Salvia* species were enriched with essential oils comprising important components: *α*-pinene, *β*-pinene, limonene, linalool, caryophyllene, germacrene, myrcene, *α*-thujone, and humulene. Potential health benefits owing to anticancer, antioxidant, antidiabetic, anti-inflammatory, antithrombotic, antirheumatic, and antiviral properties were reported from *Salvia* species. *Salvia* phytochemicals have been studied as regulating anticancer mechanisms at the cellular level by effectively modulating host cell responses in multiple ways. This review summarizes and discusses recent studies on the metabolite profiling of *Salvia* plants and bioactivities of the extracts and compounds. It may provide future perspectives on the *in silico* and pharmacognostic studies on potent *Salvia* compounds. Isolation and evaluation of bioactive compounds from the least studied species is recommended.

## Graphical Abstract

**Figure:**
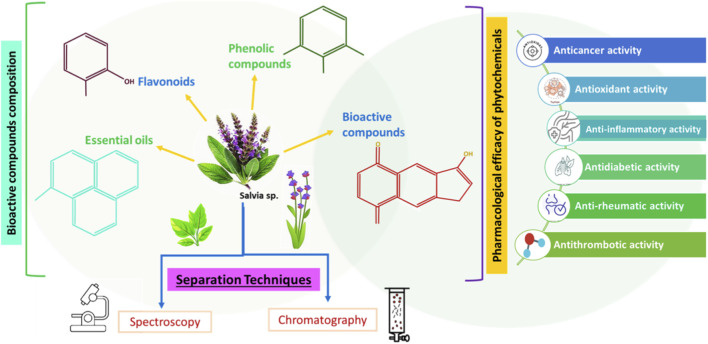

## 1 Introduction

Plants are an important source of medicine in all eras (Nwozo et al., 2023). For centuries, herbal medicines and bioactive plant compounds have been used as traditional curatives. Now, these are progressively integrated into modern medical practices (Kızıltaş et al., 2023; Bjørklund et al., 2024). Medicinal plants are widely used to treat various diseases. They possess health-promoting effects and are used to treat neurological disorders (Nasir et al., 2024), glaucoma, cancer (Samuel et al., 2024), and other diseases related to oxidative stress (Karagecili et al., 2023; Ashraf et al., 2024). It is well established that medicinal plants are a source of active compounds, including alkaloids, terpenoids, phenolic compounds, flavonoids, and fatty acids, which exhibit significant biological activity (Bingol et al., 2021; Anbessa et al., 2024). The Lamiaceae family comprises more than 7,000 species and 250 genera, including many culinary herbs such as basil, mint, rosemary, and thyme (Ali et al., 2024; Cristani and Micale, 2024). The subfamily Nepetoideae encompasses 33 genera and approximately 3,685 species. Species of this subfamily have culinary value, medicinal properties, and are ingredients in the cosmetic industry (Ortiz-Mendoza et al., 2022). *Salvia* is the largest genus of herbaceous perennials (Ihsan et al., 2024). It consists of more than 1,000 distinct species and is found on several continents across the world (Esmaeili et al., 2022; Kalnyuk et al., 2025). *Salvia* is widely distributed throughout the Mediterranean, eastern and southern Asia, and Mexico/South America (Abd Rashed and Rathi, 2021; Mátis et al., 2023; Nikolova and Aneva, 2017) mentioned in their study that 36 species in the Flora Europea belong to the genus *Salvia*. *Salvia*, derived from the Latin word “salvare” or sage, meaning “to heal”. *Salvia* species have been used since ancient times to treat a variety of diseases, including colds, cardiovascular diseases, gastric diseases, diabetes, and bronchitis (Deshmukh, 2022; Uysal et al., 2023; Kharazian et al., 2024).

**FIGURE 1:**
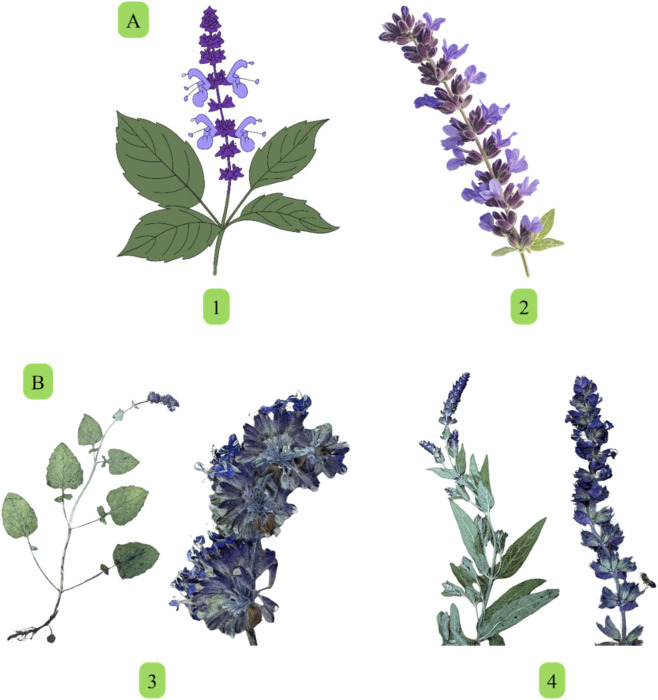
**(A)** Illustration of *Salvia miltiorrhiza* (1), and *Salvia nemorosa* (2). **(B)** Photographs of *Salvia verticillata* L. (3) and *Salvia nemorosa* L. subsp. *nemorosa* (4) from the herbarium specimens of Prof. Jarosław Proćków.

Plants are described as herbaceous and belong to all life forms (annuals, biennials, and perennials). The flowers grow in clusters and range in color from blue to red, with white and yellow less prevalent (Figure 1) (Askari et al., 2021). The stamens of *Salvia* species are described as lever-shaped stamens formed by elongated connective and stamen filaments (Kalnyuk et al., 2025). Plant species of this genus play a crucial role in traditional medicine (Li et al., 2021) and horticulture. This is due to the presence of various phytochemicals, including essential oils, phenolic compounds, and flavonoids. Promising pharmacological properties: antioxidant (Jing et al., 2023), antidiabetic (Niu et al., 2022), antimicrobial (Dembińska et al., 2025), anti-inflammatory (Liu et al., 2023), and cytotoxic properties (Demirpolat, 2023) have been reported in *Salvia* species. These effects are attributed to key compounds such as rosmarinic acid **[1]**, salvianolic acids **[146, 167, 168, 197, 199]**, camphor **[48]**, and 1,8-cineol **[43]** (Terzi et al., 2025). Additionally, the genus is economically important in aromatherapy and cosmetics due to its fragrance-rich oils. The composition of essential oils varies significantly between species and environmental conditions, making these plants a subject of ongoing phytochemical studies (Giuliani et al., 2020). For a long time, sage has been extensively used to flavor food, aromatics, and beauty products (Uysal et al., 2023).

Diverse chemical compounds and the composition of essential oils (EOs) are observed in the species of sage, such as *Salvia deserta* (desert sage), consisting of 0.02% EOs in aerial parts. In comparison, the seeds contain 23% fatty acids (Zhumaliyeva et al., 2023). *Salvia rosmarinus* Spenn. has analgesic, antitumor, and antioxidant activity, and EOs have applications in cosmetics and aromatherapy (Dejene et al., 2025). This review examines the therapeutic potential of 59 *Salvia* extracts in healthcare, identifies key chemical constituents in various *Salvia* species, and explores their mechanistic pharmacological relevance. A total of 43 *Salvia* species were reported in studies on their chemical profile. It revealed the presence of 273 bioactive metabolites belonging to the group: phenolic acids, terpenes, flavonoids, essential oils, and fatty acids. However, it is observed that 37 species of *Salvia* were evaluated for pharmacological efficacy. It includes the evaluation of their antioxidant, anticancer, antimicrobial, antidiabetic, anti-inflammatory, and neuroprotective activity. Twenty-two species were studied both for chemical profiling and biological activity. Some of these commonly analyzed *Salvia* plants were: *S. officinalis* L., *S. rosmarinus*, *S. cadmica* Bioss., *S. verticillata*, *S. nemorosa* L., *S. fruticosa* Mill., *S. verbenaca* L., *S. palaestina* Benth., *S. hispanica* L., and *S. miltiorrhiza* Bunge.

## 2 Materials and methods

A systematic approach was used to analyze, collect, and summarize recent studies and trends in research work conducted around the world on 59 *Salvia* species (Table 1). Data collection was carried out for this study, focusing on the aspects of phytochemistry and pharmacological effects of *Salvia* species. The data on ethnomedicinal use, phytochemical compounds, and pharmacological activities were collected from recent literature studies. Different databases were used to access publications: PubMed, SpringerLink, Scopus, Web of Science, ScienceDirect, and Wiley Online. For the identification of relevant publications, keywords such as “Chemical profile of *Salvia*” and “*Salvia* bioactivities” are used. Searches were made for the literature published from 2020 to 2025. Secondary metabolites of *Salvia* and therapeutic activities were taken as inclusion criteria for publications. Inclusive criteria were taxonomic, agricultural, and environmental studies on the selected genus. The naming of plants was verified using the World Flora Online portal (WFO). Their full names, with citations to their authors’ names, are given in Tables 1, 3, 4. Figures were designed using Canva Software.

**TABLE 1:** List of 59 *Salvia* species reviewed for the Phytochemical and Bioactivities Assessment.

Plant Taxa from the *Salvia* genus			
*S. officinalis* L	*S. rosmarinus*	*S. santolinifolia* Boiss	*S. macrosiphon *Boiss
*S. absconditiflora* Greuter & Burdet	*S. mirzayanii* Rech f. and Esfand	*S. compressa* Vent	*S. leucantha* Cav
*S. verbenaca* L	*S. hispanica*	*S. verticillata* L	*S. hydrangea* DC. ex Benth
*S. palaestina* Benth	*S. aethiopis *L	*S. chudaei* Batt. and Trab	*S. fructicosa* × *officinalis*
*S. fruticosa* Mill	*S. aristata* Aucher ex Benth	*S. substolonifera* E. Peter	*S. tomentosa* Mill
*S. apiana*	*S. lanceolata* Lam	*S. chamelaeagnea* Berg	*S. limbate*
*S. cadmica* Boiss	*S. aurea* L. *(S. africana-lutea* L*)*	*S. chloroleuca* Rech f. and Allen	*S. divinorum* Epling and Játiva
*S. sclarea* L	*S. ceratophylla*	*S. plebeia*	*S. balansae* Noë ex Coss
*S. potentillifolia*	*S. russellii*	*S. elegans* Vahl	*S. spinosa*
*S. pisidica*	*S. bucharica*	*S. tebesana* Bunge	*S. eremophila*
*S. multicaulis*	*S. sahendica*	*S. lachnocalyx* Hedge	*S. dominica*
*S. hierosolymitana*	*S. caespitosa*	*S. leriifolia*	*S. repens* Burch. ex. Benth
*S. barrelieri*	*S. deserta* Schangin	*S. lavandulaefolia*	*S. uliginosa* Benth
*S. aspera*	*S. nemorosa* L	*S. miltiorrhiza*	*S. cilicica* Boiss
*S. triloba*	*S. atropatana*	*S. chorassanica*	

## 3 Results and discussion

### 3.1 Phytochemical composition of *Salvia* species

Flavonoids, anthocyanins, phenolic acids, phenolic glycosides, polysaccharides, terpenoids, coumarins, and essential oils are among the main phytochemicals found in *Salvia* species (Moshari-Nasirkandi et al., 2024). The bioactivity of *Salvia* species is owed to the presence of this diverse range of chemical compounds (Bingol et al., 2021) as shown in Table 2. This table enlists 20 species and 273 compounds from these groups: phenolic acids, flavonoids, terpenoids, fatty acids, and sterols.

**TABLE 2:** Secondary metabolites reported in different parts and extracts of *Salvia* species, along with the isolation technique and analytical methods.

Plant species	Plant part/Fraction	Isolation technique	Analytical method	Compounds	References
*S. cadmica* Boiss	Aerial and roots/Hydromethanolic	Not available	UPLC-DAD/ESI-MS/MS HPLC-DAD	Rosmarinic **[1]**, salvianolic acid K **[2]**	Piątczak et al. (2021)
*S. absconditiflora* Greuter & Burdet *S. sclarea* L *S. palaestina* Benth	Aerial/Ethylacetate	Maceration	HPLC-MS/MS	Cynaroside **[3]**, rosmarinic acid **[1]**, cosmosiin **[4]**, luteolin **[5]**, apigenin **[6]**, acacetin **[7]**	Onder et al. (2022)
*S. verbenaca* L	Aerial/Essential Oils (EOs)	Steam Distillation	GC-MS	Germacrene D **[8]**, *β*-phellandrene **[9]**, *α*-copaene **[10]**, *β*-caryophyllene **[11]**, epi-*α*-cadinol **[12]**, and 1,10-di-epi-cubenol **[13]**	Belloum et al. (2014), Mrabti et al. (2022)
*S. verbenaca* L	Flower/Volatile Organic Constituents (VOCs)	HS-SPME	GC-MS	*γ*-Selinene **[14]**, germacrene D **[8]**, *β*-caryophyllene **[11]**, sabinene **[15]**, and trans-sabinene hydrate **[16]**	Al-Jaber et al. (2020), Khouchlaa et al. (2021)
*S. fruticosa* Mill	Leaves/70% ethanol	Ultrasound-assisted Extraction	LC-Q-Orbitrap HRMS	salvigenin **[17]**, apigenin **[6]**, jaceosidin **[18]**, genkwanin **[19]**, isorhamnetin **[20]**, hesperidin **[21]**	Mróz and Kusznierewicz (2023)
*S. hispanica *L	Aerial/70% ethanol	Cold Maceration	UPLC-ESI-MS/MS	*β*-sitosterol **[22]**, betulinic acid **[23]**, oleanolic acid **[24]**, *β*-sitosterol-3-*O*-*β*-D-glucoside **[25]**	Abdel Ghani et al. (2023)
*S. hispanica* L	Seeds/85% methanol	Maceration	UPLC-ESI-MS	Raffinose **[26]**, rosmarinic acid **[1]** and its derivatives, saponarin **[27]** and its isomer, Vicenin-2 **[28]**, oleic acid **[29]**, hederagenin **[30]**	Mohamed et al. (2024)
*S. hispanica* L	Seeds/ethylacetate	Maceration	GLC-MS	Palmitic acid **[31]**, *α*-Linolenic acid **[32]**, stearic acid **[33]**, *γ*-sitosterol **[34]**, and *β*-sitosterol **[22]**	
*S. macrosiphon *Boiss	Aerial/methanol	Maceration	CC, TLC, NMR	13-epi manoyl oxide **[35]**, 6-α-hydroxy-13-epimanoyl oxide **[36]**, 5-hydroxy-7,4′-dimethoxyflavone **[37]**, and *β*-sitosterol **[22]**	Balaei-Kahnamoei et al. (2021)
*S. leucantha* Cav	Aerial parts/EOs	Steam Distillation	Steam distillation GC-MS and GC-FID	6.9-guaiadiene **[38]**, *(E)*-caryophyllene **[39]**, germacrene D **[8]**, *(E)*-*β*-farnesene **[40]**, and bicyclogermacrene **[41]**	Villalta et al. (2021)
*S. hydrangea* DC. ex Benth	Leaves and flowers/EOs	Hydrodistillation	GC-MS	Spathulenol **[42]**, 1,8-cineole **[43]**, *β*-caryophyllene **[11]**, *β*-pinene **[44]**, *β*-eudesmol **[45]** in leaves, while flowers contain caryophyllene oxide **[46]**, 1,8-cineole **[43]**, *β*-caryophyllene **[11]**, *β*-eudesmol **[45]**, caryophyllenol-II **[47]**, and camphor **[48]**	Ghavam et al. (2020)
*S. officinalis* L	Ariel/EOs	Hydrodistillation	GC-MS	Naphthalenone **[49]**, camphor **[48]**, 1.8-cineole **[43]**, and *α*-thujone **[50]**	Assaggaf et al. (2022)
*S. tomentosa* Mill	Whole plant/EOs	Hydrodistillation	GC-MS and GC-FID	Camphor **[48]**, *γ*-muurolene **[51]**, *α*-pinene **[52]**, *α*-caryophyllene **[53]**, viridiflorol **[54]**, *δ*-cadinene **[55]**, and terpinene-4-ol **[56]**	Koçer and İstifli (2022)
*S. sclarea* L	Ariel/EOs	Hydrodistillation	GC-MS	Linalool acetate **[57]**, linalool **[58]**, (*E*)-caryophyllene **[39]**, *p*-cymene **[59]**, *a*-terpineol **[60]**, and geranyl acetate **[61]**	Kačániová et al. (2023), Bojan et al. (2024)
*S. officinalis* L	Ariel/EOs	Hydrodistillation	GC-MS	Camphor **[48]**, 1,8-cineole **[43]**, *β*-pinene **[44]**, camphene **[62]**, and *α*-thujone **[50]**	Tundis et al. (2020)
*S. balansae* Noë ex Coss	Leaves/Methanol	Maceration	HPLC-DAD	Luteolin **[5]**, ferulic acid **[63]**, vanillic acid **[64]**, kaempferol **[65]**, benzoic acid **[66]**, quercetin **[67]**, myricetin **[68]**, and ascorbic acid **[69]**	Mokhtar et al. (2023)
*S. chudaei* Batt. and Trab	Aerial/ethanol	Maceration	HPLC	Catechin hydrate **[70]**, resorcinol **[71]**, ferulic acid **[63]**, sinapic acid **[72]**, and resveratrol **[73]**	Tlili et al. (2025)
*S. substolonifera* E. Peter	Whole plant/95% ethanol	Maceration	CC, TLC, NMR, FTIR, HRESIMS	Substolide H **[74]**, ferruginol **[75]**, dihydrotanshinone I **[76]**, methyl rosmarinate **[77]**, ursolic acid **[78]**, caffeic acid **[79]**, and digitoflavone **[80]**	Zhong et al. (2025)
*S. santolinifolia* Boiss	Roots/90% methanol	Maceration	1 D, 2 D NMR, and HR-ESIMS	Aegyptinone E **[81]**, aegyptinone A **[82]**, and aegyptinone D **[83]**	Sargazifar et al. (2024)
*S. compressa* Vent	Shoots/dichloromethane	Maceration	CC, NMR	Citrostadienol **[84]**, *β*-sitosterol **[22]**, linolenic acid **[85]**, linoleic acid **[86]**, palmitic acid **[31]**, and geraniol **[87]**	Noorbakhsh et al. (2022)
*S. aurea* L. *(S. africana-lutea* L*)* *S. lanceolata* Lam *S. chamelaeagnea* Berg	Aerial/Methanol	Ultrasonic-assisted Extraction	UPLC-qToF-MS	Caffeic acid **[79]**, rosmarinic acid **[1]**, carnosol **[88]**, carnosic acid **[89]**, and ursolic acid **[78]**	Tock et al. (2021)
*S. officinalis *L	Leaves/water	Water bath shaking	UPLC-MS/MS	Procyandinin trimer **[91]**, epigallocatechin gallate **[92]**, epicatechin gallate **[93]**, catechin **[94]**, epicatechin **[95]**, Ruthin **[96]**, kaempferol-3-rutinoside **[97]**, quercetin-3-rhamnoside **[98]**, kaempferol-3-*o*-hexoside **[99]**, luteolin **[5]**, apigenin **[6]**, rosmarinic acid **[1]**, chlorogenic acid **[100]**, ferulic acid **[63]**, caffeic acid **[79]**, syringic acid **[101]**, gallic acid **[102]**, hydroxybenzoic acid **[103]**	Maleš et al. (2022)
*S. hispanica*	Seed/oil	Orbital Shaking	HPLC	Rosmarinic acid **[1]**, chlorogenic acid **[100]**, gallic acid **[102]**, and caffeic acid **[79]**	Gebremeskal et al. (2024), Mutlu et al. (2025)
*S. hispanica*	Seed/oil	Cold Pressing	LC-HRMS	Ascorbic acid **[69]**, chlorogenic acid **[100]**, caffeic acid **[79]**, rosmarinic acid **[1]**, ellagic acid **[104]**, salicylic acid **[105]**, hispidulin **[106]**, and luteolin **[5]**	Mutlu et al. (2025)
*S. aethiopis *L	Ariel/ethanol	Orbital Shaking	LC-MS/MS	Hydroxybenzoic acid **[103]**, caffeic acid **[79]**, ellagic acid **[104]**, *p*-Coumaric acid **[107]**, rosmarinic acid **[1]**, syringic acid **[101]**, chlorogenic acid **[100]**, ferulic acid **[63]**, hyperoside **[108]**, hesperidin **[21]**, rutin **[109]**, luteolin **[5]**, kaempferol **[65]**, quercetin **[67]**, apigenin **[6]**, galangin **[110]**, naringenin **[111]**, and genkwanin **[10]**	Bilginoğlu et al. (2025)
*S. aristata* Aucher ex Benth	Aerial/EOs	Hydrodistillation	GC-MS	*β*-caryophyllene **[11]**, caryophyllene oxide **[46]**, bicyclogermacrene **[41]**	Dabaghian et al. (2025)
*S. officinalis* L	Aerial/ethanol	Maceration	LC-ESI-MS	Cirsilineol **[113]**, cirsiliol **[114]**, luteolin **[5]**, apigenin **[6]**, naringenin **[111]**, kaempferol **[65]**, quercetin **[67]**, naringin **[115]**, apigenin-7-*o*-glucoside, **[116]**, luteolin-7-*o*-glucoside **[117]**, rosmarinic acid **[1]**, syringic acid **[101]**, *p*-coumaric acid **[107]**, caffeic acid **[79]**, protocatechuic acid **[118]**, chlorogenic acid **[100]**, quinic acid **[119]**, and ferulic acid **[63]**	Akrimi et al. (2025)
*S. officinalis* L	Leaves/EOs	HS-SPME	GC-MS	Hexanal **[120]**, *trans*-salvene **[121]**, *cis*-salvene **[122]**, tricyclene **[123]**, camphene **[62]**, *α*-thujene **[124]**, *α*-pinene **[52]**, *β*-pinene **[44]**, sabinene **[15]**, 1-octen-3-ol **[125]**, *α*-phellandrene **[126]**, *α*-terpinene **[127]**, *γ*-terpinene **[128]**, *p*-cymene **[59]**, eucalyptol **[130]**, cis-sabinene hydrate **[131]**, cis-linalool oxide **[132]**, linalool **[58]**, terpinolene **[133]**, *α*-thujone **[50]**, *β*-thujone **[134]**, *α*-campholenal **[135]**, camphor **[48]**, iso-thujol **[136]**, humulene-1,2-epoxide **[137]**, viridiflorol **[54]**, *β*-caryophyllene **[11]**, and *α*-humulene **[138]**	Pachura et al. (2022)
*S. deserta *Schangin	Roots/95% ethanol	Maceration	HPLC	Salvidesertone A **[139]**, Salvidesertone B **[140]**, Salvidesertone C **[141]**, 8*α*,9*α*-epoxy-7-oxoroyleanone **[142]**, 8*α*,9*α*-epoxy-6-deoxycoleon U **[143]**	Zheng et al. (2020)
*S. verticillata* L	Aerial/Methanol	Maceration	UPLC/MS-MS	Salvianic acid A **[144]**, caffeic acid **[79]**, coumaric acid **[145]**, salvianolic acid C **[146]**, dicaffeoylquinic acid **[147]**, hydroxyrosmarinic acid **[148]**, rosmarinic acid **[1]**, quercetin 3-*O*-rutinoside **[149]**, luteolin 7-*O*-glucoside **[117]**, luteolin 7-*O*-hexuronide **[150]**, quercetin 3-*O*-rhamnoside **[151]**, apigenin 7-*O*-glucoside **[116]**, apigenin 7-*O*-hexuronide **[152]**, and apigenin **[6]**	Stanković et al. (2020)
*S. verbenaca *L	Aerial/EOs	Steam Distillation	GC-MS	*α*-pinene **[52]**, *β*-pinene **[44]**, sabinene **[15]**, 1,8-cineole **[43]**, *β*-phellandrene **[9]**, linalool **[58]**, *p*-cymene **[59]**, linalyl acetate **[153]**, E-*β*-ocimene **[154]**, (Z)-*β*-ocimene **[155]**, tricyclene **[123]**, camphor **[48]**, 1,10-di-epi-cubenol **[13]**, epi-13-manool **[156]**, cis-muurola-3,5-diene **[157]**, *γ*-selinene **[14]**, trans-sabinene hydrate acetate **[158]**, *β*-caryophyllene **[11]**, viridiflorol **[54]**, and germacrene D **[8]**	Aissaoui et al. (2014), Mrabti et al. (2022)
*S. mirzayanii* Rech f. and Esfand	Seeds/80% methanol	Maceration	GC-MS	Linalool **[58]**, spathulenol **[42]**, *δ*-cadinene **[55]**, cubenol **[159]**, *β*-eudesmol **[45]**, *α*-cadinol **[160]**, linalyl acetate **[153]**, and *α*-terpinyl acetate **[161]**, teuclatriol **[162]**, bicyclogermacrene **[41]**, chrysoeriol **[163]**, cirsimaritin **[164]**, salvigenin **[17]**	Shahraki et al. (2024)
*S. nemorosa L*	Aerial/80% Methanol	Ultrasonic bath	UHPLC-HRMS	Rosmarinic acid **[1]**, ferulic acid **[63]**, caffeoylquinic acid **[165]**, syringic acid **[101]**, sagerinic acid **[166]**, salvianolic acid A **[167]**, salvianolic acid B **[168]**, salvianolic acid C **[146]**, salvianolic acid K **[2]**, yunnaneic acid F **[169]**, yunnaneic acid E **[170]**, caffeic acid **[79]**, sagecoumarin **[171]**, verbascoside **[172]**, forsythoside A **[173]**, myricitrin **[174]**, hyperoside **[108]**, eriodictyol-*O*-glucuronide **[175]**, hispidulin **[106]**, genistein **[176]**, 6-hydroxyluteolin 7-*O*-glucuronide **[177]**, lipedoside A **[178]**, luteolinglucoside **[179]**, luteolin **[5]**, luteolinglucuronide, apigenin 7-*O*-glucoside **[180]**, apigenin **[6]**, kaempferol **[65]**, luteolin acetyl glucoside **[181]**	Moshari-Nasirkandi et al. (2024)
*S. miltiorrhiza Bunge*	Roots/Methanol	Water bath	UHPLC	Tanshinone I **[182]**, cryptotanshinone **[183]**	Tran et al. (2024)
*S. elegans* Vahl	Aerial/ethylacetate	Maceration	HPLC	*a*-amirin **[184]**, *b*-amirin **[185]**, ursolic acid **[78]**, oleanolic acid **[186]**, corosolic acid **[187]**, maslinic acids **[188]**, and eupatorine **[189]**	Gutiérrez-Román et al. (2022)

#### 3.1.1 Non-volatile compounds present in *Salvia* species

The storage of non-volatiles was observed in plant parts such as flavonoids, triterpenoids, monoterpenoids, and sesquiterpenoids in the above-ground portion, while roots accumulate diterpenoids and phenolic acids. These compounds are believed to impart useful properties of promoting health and healing (Askari et al., 2021). Different groups of non-volatiles are present in the species of sage: terpenoids (diterpenes, triterpenes, sisterpenes), flavonoids (flavanols, flavonols, flavones), caffeic acid derivatives, and phenolic acids (Tock et al., 2021; Maciel et al., 2022; Maleš et al., 2022; Luca et al., 2023). More than 160 polyphenols have been identified in *Salvia* species. Caffeic acid occurs mainly in the dimer form, as rosmarinic acid **[1]**, in the Lamiaceae family. It is a building block for a variety of plant metabolites, from monomers to oligomers, and a large number of polyphenolic compounds are constructed from it through various condensation reactions (Zhumaliyeva et al., 2023).

**FIGURE 2:**
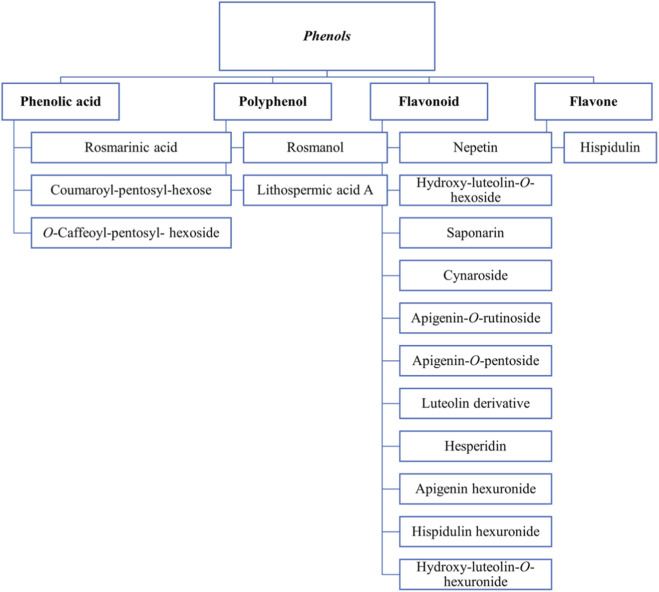
Different classes of phenols present in *Salvia* species.

Natural phenolic compounds exhibit many beneficial health effects in humans (Saleem et al., 2022), including antioxidant, antimicrobial (Dembińska et al., 2025), anticancer (Kapil et al., 2025), and anti-inflammatory activity (Liu et al., 2023). Lamiaceae and its largest genus, *Salvia,* are among the richest sources of antioxidant and antimicrobial phenolics (Piątczak et al., 2021). In a study by Onder et al. (2022), acacetin **[7]** was the highest phenolic compound in the extracts of *S. sclarea* and *S. palaestina* (24.094 and 69.297 mg analyte/g of dry extract, respectively). The total flavonoid content was 83.23, 60.62, and 58.71 mg RE/g of extract in *S. palaestina*, *S. absconditiflora,* and *S. sclarea*, respectively. Righi et al. (2021) quantified phenolics in the extract of *S. verbenaca,* and the amount was 206 mg GAE/g extract. Moshari-Nasirkandi et al. (2024) analyzed the TPC and TFC of 102 samples from 20 species of *Salvia*. The maximum TPC of >55 mg GAE/g DW was shown by samples of *S. ceratophylla*, *S. verticillata*, *S. nemorosa,* and *S. limbata*. These species were also rich in TFC. Nilofar et al. (2024) reported a TPC of 92.10 mg GAE/g and a TFC of 50.85 mg RE/g in the *Salvia* hybrid (*S. fructicosa* × *officinalis*), providing the plant with promising antioxidant activity. Some of the phenols listed in the study are shown in Figure 2 (Nilofar et al., 2024).

Khouchlaa et al. (2021) enlisted the main phenolics in *S. verbenaca,* such as: rosmarinic acid **[1]**, p-hydroxybenzoic acid **[190]**, and caffeic acid **[79]**. They reported the presence of four flavonic aglycons (apigenin **[6]**, luteolin **[5]**, salvigenin **[17]**, and 5-hydroxy-7,4′-dimethoxyflavone **[37]**) in the leaves of *S. verbenaca* from Spain and three flavonoids (5-hydroxy-3,4′,7-trimethoxyflavone **[191]**, retusin **[192]**, verbenacoside **[193]**) in aerial parts of samples collected from Saudi Arabia. Naringenin **[111]**, hesperidin **[21]**, and cirsiliol **[114]** were the main flavonoids identified in the flowering stage of Tunisian species.

Rashwan et al. (2021) identified 12 polyphenols by HPLC analysis of *S. officinalis* EOs. The main compounds were coumaric acid **[145]** (0.043 mg/g), chlorogenic acid **[100]** (0.037 mg/g), caffeic acid **[79]** (0.028 mg/g), catechin **[94]** (0.025 mg/g), vanillin **[194]** (0.024 mg/g), ellagic acid **[104]** (0.019 mg/g), gallic acid **[102]** (0.017 mg/g) and naringenin **[111]** (0.011 mg/g). Francik et al. (2020) studied the phenolic composition of *S. officinalis* dried in different ways. Ferulic acid **[63]**, rutin **[109]**, hesperidin **[21]**, catechin **[94]**, quercetin **[67]**, isorhamnetin **[20]**, 3,5-dicaffeoylquinic acid **[195]**, *p*-coumaric acid **[107]**, and sinapinic acid **[196]** were reported in all samples. Maciel et al. (2022) reported the presence of caffeic acid **[79]**, rosmarinic acid **[1]**, salvianolic acid I **[197]**, methyl salvianolate I **[198]**, salvianolic acid K **[2]**, salvianolic acid L **[199]**, and sagerinic acid **[166]** in *S. officinalis*. Krol et al. (2022) observed the accumulation of different phenolics in *S. apiana.* The ethanol extract contained cirsimaritin **[164]** and salvigenin **[17]**, whereas in the decoction, hesperidin **[21]**, quercetin-*O*-hexoside **[200]**, and cirsimaritin **[164]** were observed. Rosmarinic acid **[1]** was determined in methanol extracts (1.1 mg/mL).

Phenolic acids and tanshinones are the main bioactive ingredients of *S. miltiorrhiza*. Pharmacologically active phenolics are rosmarinic acid **[1]** and salvianolic acid B **[168]**, which play a role in anticoagulation, antioxidation, and antithrombosis (Deng et al., 2020; Tran et al., 2024). Park et al. (2025) reported phenolic acids such as caffeic acid **[79]**, sinapic acid **[72]**, benzoic acid **[66]**, ferulic acid **[63]**, rosmarinic acid **[1]**, *trans*-cinamic acid **[202]**, salvianolic acid A **[167]**, and salvianolic acid B **[168]** in the ethanol extract of *S. miltiorrhiza*.

Mokhtar et al. (2023) identified catechin **[94]** (72.5%), myricetin **[174]** (21.7%), epicatechin **[95]** (1.3%), butylated hydroxyanisole **[201]** (1.1%) as the main phenolics in *S. balansae* by HPLC-MS. Motyka et al. (2022) reported the presence of polyphenols in the seeds of *S. hispanica,* such as gallic acid **[102]**, caffeic acid **[79]**, ferulic acid **[63]**, *p*-coumaric acid **[107]**, chlorogenic acid **[100]**, rosmarinic acid **[1]**, apigenin **[6]**, kaempferol **[65]**, quercetin **[67]**, myricetin **[68]**, rutoside **[203]**, daidzein **[204]**, glycitin **[205]**, genistein **[176]**, genistin **[206]**, and epicatechin **[95]**.

Karadeniz-Pekgöz et al. (2024) analyzed phenolic acids and flavonoids by HPLC–DAD in seeds of four *Salvia* species. *Salvia potentillifolia* was enriched with caffeic acid **[79]** (0.02 mg/mL), rosmarinic acid **[1]** (0.01 mg/mL), and quercetin **[67]** (0.09 mg/mL) were found in *S. pisidica*. *Salvia cadmica* was highly rich in caffeic acid **[79]** (0.01 mg/mL) and quercetin **[67]** (0.05 mg/mL). Ferulic acid **[63]** (0.01 mg/mL) and rutin **[109]** (0.02 mg/mL) were observed in *S. hispanica*.

**FIGURE 3:**
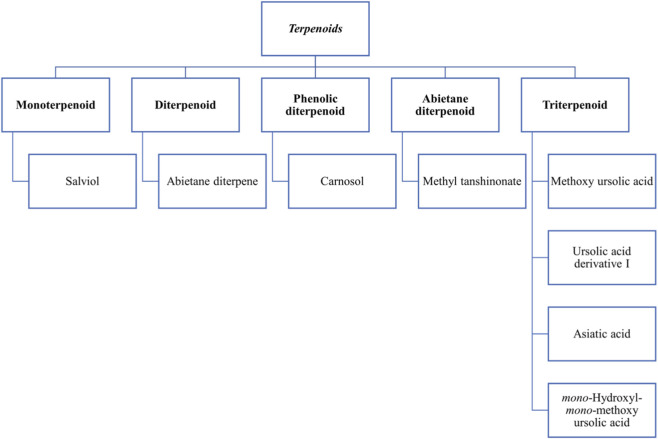
Diversity of terpenoid classes reported in *Salvia* species.

Five classes of terpenoids were reported in *Salvia* species (Figure 3) (Nilofar et al., 2024). Sargazifar et al. (2024) mentioned in their study that abietane diterpenoids are the largest class of compounds in the *Salvia* genus. Out of the 545 known *Salvia* diterpenoids, 365 are abietane diterpenoids. Three 20,24-epoxydammarane triterpenes (santolin A, santolin B, and avinsl C **[207–209]**), two amyrin-type triterpenes (slavins A and B **[210–211]**), and a new ursane-type triterpene (santolinoic acid **[212]**) have been isolated and identified from *S. santolinifolia*. Another study by Maciel et al. (2022) reported the presence of diterpenes in *Salvia* species; 12-methoxy-carnosic acid **[213]** in *S. repens* Burch. ex. Benth and isoicetexone **[214]**, icetexone **[215]** in *S. uliginosa* Benth. They also reported triterpenes (ursolic acid **[78]**, oleanolic acid **[186]**) in *S. cilicica* Boiss.

Khouchlaa et al. (2021) evaluated the terpenoid profile of the petroleum ether extract of the roots of *S. verbenaca*. The distribution of compounds was as follows: roots having taxodione **[216]**, horminone **[217]**, and 613-hydroxy-7-acetoxyroyleanon **[218]**. The presence of epi-13-manool **[156]** and manool **[219]** was observed in leaves, *β*-caryophyllene **[11]** and caryophyllene oxide **[46]** in fruits, and stems enriched with camphor **[48]** and viridiflorol **[54]**. The main phenolic diterpenoids were methyl carnosate **[220]** and carnosic acid **[89]**.

Krol et al. (2022) reported the presence of triterpenes (oleanolic acid **[186]**, ursolic acid **[78]**, uvaol **[221]**) and diterpenes (sageone **[222]**, carnosol **[88]**, 16-hydroxycarnosol **[223]**, rosmadial **[224]**) in the aerial part of ethanol extracts of *S. apiana*. Different diterpenes were found in the acetone extract, such as 16-hydroxycarnosic acid **[225]**, salvicanol **[226]**, rosmanol **[227]**, 7-epirosmanol **[228]**, 16-hydroxycarnosol **[223]**, 16-hydroxyrosmanol **[229]**, and 16-hydroxy-7-methoxyrosmanol **[230]**. However, the composition of the leaf extract was diterpenes (carnosic acid **[89]**, carnosol **[88]**, 16-hydroxycarnosic acid **[225]**) and triterpenes (*α*-amyrin **[184]**, oleanolic acid **[186]**, ursolic acid **[78]**). Zhong et al. (2025) isolated and identified a new compound using different approaches, such as the HR-ESIMS, COSY, and NOESY experiments in conjunction with ECD analysis in *S. substolonifera*. The name of the compound is substolide H **[74]** and is a norditerpene lactone. Barhoumi et al. (2022) reported a total of 14 compounds from the aqueous methanol fraction of *S*. *multicaulis,* including a new abietane diterpene derivative identified as 2,20-dihydroxyferruginol **[231]**.

Park et al. (2025) reported the presence of dihydrotanshinone I **[76]**, cryptotanshinone **[183]**, and tanshinone IIA **[232]** in the ethanol extract of *S. miltiorrhiza* at varying concentrations in different cultivars ranging from 32 to 272 mg/100 g. Hafez Ghoran et al. (2022) listed 106 unusual terpenoids from different *Salvia* species. They reported the presence of salvilymitol **[233]** and salvilymitone **[234]** in the acetone extract of the aerial parts of *S. hierosolymitana*. Pixynol **[235]** was found in the acetone extract of the roots of *S. barrelieri*. Amblyol **[236]** and amblyone **[237]** were identified in the acetone extract of the aerial parts of *S. aspera*. Russelliinosides **[238]** are reported from the dichloromethane extract of the aerial parts of *S. russellii*. Salvadione A **[239]** and salvadione B **[240]** were isolated from the n-hexane-soluble fraction of *S. bucharica*. The n-hexane extract from aerial parts of *S. hydrangea* was enriched with salvadione C **[241]**, perovskone B **[242]**, and hydrangenone **[243]**. A new norsesterterpene (C-17, C-18, C-19, and C-20 tetranorsesterterpene **[244]**) was isolated from the acetone extract of the aerial parts of *S. sahendica*.

#### 3.1.2 Essential oils

Essential oils (EOs) contain VOCs of various functional groups (Levaya et al., 2025). EOs are a rich source of bioactive compounds and may contain up to 200 compounds (Pezantes-Orellana et al., 2024). Many *Salvia* species are known for their essential oils, which are primarily composed of four chemotypes, with sesquiterpenes being the most common, particularly *β*-caryophyllene **[11]** and germacrene D **[8]** (Asgarpanah, 2021). Monoterpenes in *Salvia* oils are: *α*-pinene **[52]**, *β*-pinene **[44]**, camphor **[48]**, limonene **[245]**, linalool **[58]**, and borneol **[246]**. The chemical composition of the essential oil of *S. rosmarinus* from Italy is characterized mainly by monoterpene hydrocarbons (39.32%–40.70%) and oxygenated monoterpenes (36.08%–39.47%). Representative compounds are: 1,8-cineole **[43]**, *α*-pinene **[52]**, camphor **[48]**, and *β*-caryophyllene **[11]** (Leporini et al., 2020). A total of 42 compounds were analyzed using GC-MS and GC-FID in the EOs of *S. leucantha* Cav. Six major compounds were: 6.9-guaiadiene **[38]** (19.14%), *(E)*-caryophyllene **[39]** (16.80%), germacrene D **[8]** (10.22%), *(E)*-β-farnesene **[40]** (10%), bicyclogermacrene **[41]** (7.52%), bornyl acetate **[112]** (14.74%), and *α*-pinene **[52]** (3.31%) (Villalta et al., 2021). The EOs reported in the leaves of *S*. *hydrangea* were found to be predominantly composed of spathulenol **[42]** (16.07%), 1,8-cineole **[43]** (13.96%), *β*-caryophyllene **[11]** (9.58%), *β*-pinene **[44]** (8.91%), and *β*-eudesmol **[45]** (5.33%). In comparison, the EOs obtained from the flowers were characterized by a high proportion of caryophyllene oxide **[46]** (35.47%), followed by 1,8-cineole **[43]** (9.54%), *β*-caryophyllene **[11]** (6.36%), *β*-eudesmol **[45]** (4.11%), caryophyllenol-II **[47]** (3.46%), and camphor **[48]** (3.33%) (Ghavam et al., 2020).

Gourich et al. (2022) examined the EOs composition of *S. officinalis* by GC-MS, which was extracted using hydrodistillation. It showed the presence of oxygenated monoterpenes and sesquiterpenes. The main constituents are 1,8-cineole **[43]** (16.8%), *β*-thujone **[134]** (15.9%), *β*-caryophyllene **[11]** (12.6%), and camphor **[48]** (11.7%). Other compounds with a composition of 7%–8% were: *α*-humulene **[138]**, *α*-pinene **[52]**, and viridiflorol **[54]**. Some minor compounds (camphene **[62]**, *α*-thujone **[50]**, limonene **[245]**, and *α*-pinene **[52]**) were found, ranging between 1% and 3%. Rashwan et al. (2021) in another study, identified 39 components in the EOs of the *S. officinalis* aqueous extract. The main constituent was 9-octadecenamide **[247]** (55.8%), while other compounds were eucalyptol **[130]**, trimethylsaline (TMS) derivatives of palmitic acid **[31]** and stearic acid **[33]**, and other long-chain hydrocarbons and fatty acid derivatives in smaller amounts.

In line with these findings on *S. officinalis*, another study has also reported a consistent presence of oxygenated monoterpenes and sesquiterpenes as the main constituents of the EOs of *S. officinalis*. The principal compounds identified by GC-MS included naphthalenone **[49]**, camphor **[48]**, 1,8-cineole **[43]**, *α*-pinene **[52]**, camphene **[62]**, isoborneol **[248]**, and *α*-thujone **[50]** (Assaggaf et al., 2022). Similarly, in the Italian species of *S. officinalis,* camphor **[48]** (16.16%–18.92%), 1,8-cineole **[43]** (8.80%–9.86%), *β*-pinene **[44]** (3.08%–9.14%), camphene **[62]** (6.27%–8.08%), and *α*-thujone **[50]** (1.17%–9.26%) are identified as the most abundant constituents of EOs (Tundis et al., 2020). Similarly, *α*-thujone **[50]** (33.77%), *β*-caryophyllene **[11]** (12.28%), *α*-humulene **[138]** (12.19%), camphor **[48]** (11.52%), naphthalene **[249]** (9.94%), eucalyptol **[130]** (8.11%), *α*-pinene **[52]** (3.31%), *β*-pinene **[44]** (1.8%), *β*-myrcene **[250]** (1.49%), germacrene D **[8]** (1.36%), and borneol **[246]** (1.18%) were identified as the main components in other EOs of *S. officinalis* (Al-Mijalli et al., 2022).

A total of 60 compounds, accounting for 98.2% of the EOs composition, were identified in *S*. *tomentosa*. The predominant constituents included camphor **[48]** (9.35%), *γ*-muurolene **[51]** (8.37%), *α*-pinene **[52]** (7.59%), *α*-caryophyllene **[251]** (6.25%), viridiflorol **[54]** (5.13%), δ-cadinene **[55]** (5.01%), and terpinene-4-ol **[56]** (5.01%) (Koçer and İstifli, 2022). On the contrary, the EOs of *S*. *sclarea* were characterized mainly by linalool acetate **[57]** (49.1%) and linalool **[58]** (20.6%), with other notable components such as (*E*)-caryophyllene **[39]** (5.1%), *p*-cymene **[59]** (4.9%), *α*-terpineol **[129]** (4.9%), and geranyl acetate **[61]** (4.4%) (Kačániová et al., 2023). GC-MS analysis revealed the presence of 139 compounds in the EOs of 12 native Iranian *Salvia* species. Some of the common compounds reported in all samples were as follows: Linalool **[58]**, *α*-terpineol **[129]**, *β*-caryophyllene **[11]**, spathulenol **[42]**, and caryophyllene oxide **[46]**. The yield of EOs extracted from plants was also calculated in the range of 0.06%–0.96% w/w (Gharehbagh et al., 2023).

Alves-Silva et al. (2023) performed hydro distillation of *S. aurea* leaves and collected EOs. They studied the composition of EOs by GC-MS and GC-FID. Similar compounds were observed in the EOs of other *Salvia* species. The main components of EOs were: 1,8-cineole **[43]** (16.7%), *β*-pinene **[44]** (11.9%), *α*-thujone **[50]** (10.5%), camphor **[48]** (9.5%), and (*E*)-caryophyllene **[39]** (9.3%). Other compounds were limonene **[245]**, viridiflorol **[54]**, *γ*-muurolene **[51]**, *α*-humulene **[138]**, *β*-myrcene **[250]**, and *α*-pinene **[52]**.

#### 3.1.3 Fatty acids

The fatty acid profiling of seven species of *Salvia* revealed the presence of compounds of therapeutic value. Dihydroxyoctadecadienoic acid **[252]** and 13-hydroxy-9,11-octadecadienoic acid **[253]** were observed in *S. fruticosa* by (Mróz and Kusznierewicz, 2023). Mokhtar et al. (2023) reported that out of the 17 compounds, palmitic acid **[31]** was the main fatty acid, followed by oleic acid **[29]**, linoleic acid **[86]**, stearic acid **[33]**, eicosanoic acid **[254]**, and dimethyl phthalate **[255]** in the petroleum ether extract of *S. balansae*. Abdel Ghani et al. (2023) reported that the GLC-MS analysis of the seed oil of *S. hispanica* L. showed a high concentration of omega-3 fatty acids, with a percentage of 35.64% of the total fatty acid content in the seed oil. The identified compounds were methyl esters of linoleic acid **[86]** (35.64%), α-linolenic acid **[32]** (23.95), palmitic acid **[31]** (14.12%), stearic acid **[33]** (7.63%), lauric acid **[256]** (5.87%), myristic acid **[257]** (2.31%), 11,14,17-eicosatrienoic acid **[258]** (0.59%), arachidic acid **[259]** (0.57%), caprylic acid **[260]** (0.54%) and capric acid **[261]** (0.42%).

Motyka et al. (2022) reported that the oil obtained from *S. hispanica* seeds accounts for 30%–33% of the fatty acids, of which 80% are essential fatty acids (EFAs) such as α-linolenic **[32]** and linoleic acid **[86]**. Chia seeds also contain the following sterols in small amounts: campesterol **[262]** (472 mg/kg), stigmasterol **[263]** (1,248 mg/kg), *β*-sitosterol **[22]** (2057 mg/kg), and stigmasta-5,24 (28)-dien-3*β*-ol (Δ^5^-avenasterol) **[264]** (355 mg/kg). Gebremeskal et al. (2024) analyzed the fatty acid composition of *S. hispanica* seed oil by GC-MS. α-linolenic acid **[32]** was found to be the main compound with a percentage of 62.65, and other compounds were linoleic acid **[86]** (19.27%), oleic acid **[29]** (7.47%), palmitic acid **[31]** (8.79%), stearic acid **[33]** (1.66%), and myristic acid **[257]** (0.16%). Karadeniz-Pekgöz et al. (2024) determined the fatty acid profile of seeds of *Salvia* species by GC-MS. Palmitic acid **[31]**, stearic acid **[33]**, oleic acid **[29]**, and linoleic acid **[86]** were present in significant amounts in *S. cadmica*, *S. caespitosa*, *S. pisidica*, *S. potentillifolia,* and *S. hispanica*, with a maximum composition of linoleic acid [86] that was above 70% in all samples.

### 3.2 Pharmacological activities of the plant extracts of *Salvia* species

**FIGURE 4:**
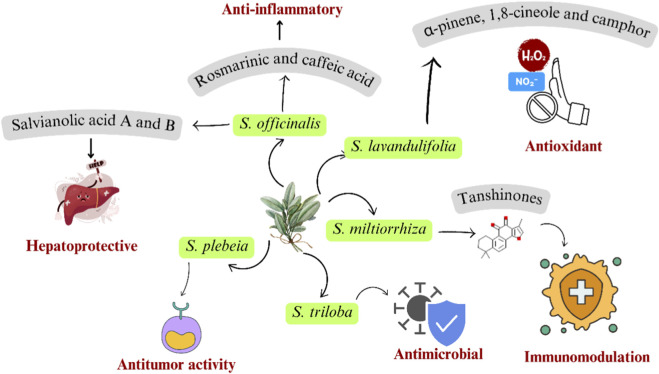
Pharmacological activity of some *Salvia* species.

Sage plants have been used for centuries in the culinary, cosmetic, and fragrance industries. It is used to cure a wide range of ailments, including digestive, respiratory, renal, hepatic, neurological, cardiac, blood circulation, and metabolic disorders (Afonso et al., 2021). The *Salvia* genus contains flavonoids, phenolic acids, terpenoids, lipophilic diterpenoids, and tanshinone derivatives. The mentioned compounds exhibit antioxidant, antibacterial, anticancer, antimicrobial, anti-inflammatory, anti-dermatophyte, antiviral, antineoplastic, and anti-platelet aggregation properties, as shown in Figure 4 (Yilmaz et al., 2022; Zhumaliyeva et al., 2023). *Salvia officinalis* is an important medicinal and aromatic plant because of its bioactive components. These components are phenolics, terpenoids, polyphenols, and flavonoids. It has an anticancer, antimicrobial, and anti-inflammatory role (Poulios et al., 2020). Some of the *Salvia* species with potential pharmacological activities are listed in Table 3.

**TABLE 3:** The therapeutic potential of various species of the genus *salvia*.

Species	Plant part/Extract	Therapeutic potential	References
*S. deserta *Schangin	Roots/Ethylacetate	Antimicrobial, antileishmanial, and antithrombotic	Zhumaliyeva et al. (2023)
*S. sclarea *L	Leaves/EOs	Antioxidant, antibacterial	Stanciu et al. (2022)
*S. verticillata* L	Leaves/EOs	Improve liver fibrosis, cardio- and hepatoprotection, Alzheimer’s disease, antioxidant potential, anti-inflammatory, antibacterial, and antifungal activity	Ivanova et al. (2024)
*S. officinalis* L	Aerial/Aqueous	Photoprotective, antioxidant, and cytotoxic activity	Tsitsigianni et al. (2023), Akacha et al. (2024)
*S. divinorum* Epling and Játiva	Leaves/Aqueous	Psychoactive	Brito-da-Costa et al. (2021), Ertas et al. (2023)
*S. absconditiflora* Greuter & Burdet	Aerial/EOs	Antioxidant, cytotoxic, and antimicrobial	Demirpolat (2023)
*S. verbenaca *L	Leaves/decoction	Antidiabetic, antipyretic, wound healing, cure skin, digestive, and respiratory problems	Mrabti et al. (2022)
*S. mirzayanii* Rech f. and Esfand	Seeds/80% methanol	Antioxidant and antibacterial Gastrointestinal diseases, skin infections, spasms, inflammations, and weakness	Hadkar and Selvaraj (2023), Shahraki et al. (2024)
*S. chloroleuca* Rech f. and Allen	Not available	Antibacterial, antitumoral, antiviral, antifungal, antiparasitic, antirheumatic, anticancer, and neuroprotective	Salimikia and Mirzania (2022)
*S. nemorosa* L	Aerial/80% ethanol	Antimicrobial, anticancer, and antioxidant	Luca et al. (2023)
*S. miltiorrhiza* Bunge	Herbal extract	Improve blood circulation, treat insomnia, abdominal and chest lumps, palpitations, and skin carbuncles	Nwafor et al. (2021), Huang et al. (2024)
*S. aurea* L. (*S. africana-lutea* L.)	Leaves/aqueous	Cold, flu, tuberculosis, headaches, fever, and chronic bronchitis	Ezema et al. (2024)
*S. elegans* Vahl	Aerial/ethyl acetate	Antihypertensive effect	Gutiérrez-Román et al. (2022)
*S. hispanica* L	Seeds/aqueous	Hypoglycemic, antimicrobial, anticancer, anti-inflammatory, antioxidant, antihypersensitive, anti-obesity, and cardioprotective properties	Amtaghri and Eddouks (2023), Ashish et al. (2022)
*S. miltiorrhiza* Bunge	Tanshinones	Antifibrotic, antitumor, and inflammatory, neuroprotection, and cardiovascular diseases	Shou et al. (2025)
*S. aethiopis *L	Aerial/aqueous	Anticancer and antioxidant	Tasheva et al. (2025)

#### 3.2.1 Antioxidant activity

Sage (*S. officinalis*) is a fragrant and medicinal herb well known for its pharmacological characteristics. DPPH, FRAP, and ABTS tests demonstrated that the best antioxidant activity of *S. officinalis* EOs was observed in the full flowering stage. IC_50_ values were 0.011 ± 3.29, 0.012 ± 2.17, and 0.014 ± 1.81 mg/mL for the DPPH, FRAP, and ABTS assays, respectively (Assaggaf et al., 2022). An antioxidant activity was found in a concentration-dependent manner for the EOs of the aerial parts of *S. officinalis.* The ABTS assay showed the highest radical scavenging with IC_50_ values of 20.64 μg/mL (Tundis et al., 2020). Another study by Al-Mijalli et al. (2022) analyzed the antioxidant activity of EOs from *S. officinalis,* and the reported results were significant. The IC_50_ values were 0.093 ± 2.17 mg/mL, 0.0112 ± 3.18 mg/mL, and 0.0129.74 ± 2.11 mg/mL for the DPPH, FRAP, and ABTS assays, respectively.

Piątczak et al. (2021) determined the antioxidant activities of hydromethanolic extracts of the aerial parts and root of *S. cadmica*. The aerial parts showed a strong antioxidant potential for DPPH with an IC_50_ value of 0.034 mg/mL. Onder et al. (2022) analyzed the antioxidant potential of the ethyl acetate extract from the aerial parts of *S. absconditiflora, S. sclarea*, and *S. palaestina*. *Salvia absconditiflora* showed significant antioxidant activity with a value of 251.39 mg TE/g extract using the DPPH assay. Mróz and Kusznierewicz (2023) showed the antioxidant activity of the 70% ethanol extract of *S. fruticosa.* It was found to be dose-dependent in the ABTS assay, as well as in the DPPH test. It is attributed to the presence of rosmarinic acid **[1]**.

Abdel Ghani et al. (2023) studied that the dichloromethane fraction of the aerial parts of *S. hispanica* revealed antioxidant activity against the DPPH radical (IC_50_ = 0.014 mg/mL). This activity was approximately comparable to that of ascorbic acid **[69]** (IC_50_ = 0.012 mg/mL). Gebremeskal et al. (2024) reported that the chia seed extract with an ethanol concentration of 80% scavenged DPPH with a maximum inhibition percentage of 90%. This activity is attributed to the presence of a flavonoid content (1.08 ± 0.20 mg QE/g extract). Akrimi et al. (2025) analyzed the antioxidant activity of the hexane extract of *S. officinalis*. The lowest IC_50_ value of 0.03 mg/mL was observed by the DPPH assay and a similar trend was shown for the FRAP assay, with the reducing power of EC_50_ = 0.17 mg/mL. An increased antioxidant activity of *S. mirzayanii* was observed after treatment with elicitors (salicylic acid **[105]** and yeast extract) (Shahraki et al., 2024).

Karadeniz-Pekgöz et al. (2024) reported that the methanol extract of *S. pisidica* showed a significant DPPH radical scavenging activity with an IC_50_ value of 0.0182 mg/mL, and it is attributed to the presence of TPC of 0.0176 mg/mL GAE. Tsakni et al. (2025) showed that the leaf extract of *S. rosmarinus* demonstrated promising antioxidant activity with the IC_50_ value of 0.0129 mg/mL. It is attributed to the synergistic effect of phenolic acids such as rosmarinic acid **[1]**, benzoic acid **[66]**, and vanillic acid **[64]**.

#### 3.2.2 Anti-inflammatory properties

Assaggaf et al. (2022) showed that *S. officinalis* EO at the full flowering stage exhibits the best anti-inflammatory activity with an IC_50_ value of 0.092 ± 0.03 mg/mL, while quercetin **[67]** activity was IC_50_ = 0.048 ± 0.02 mg/mL. Righi et al. (2021) showed that the hydromethanolic extract of *S. verbenaca* exhibited a strong anti-lipid peroxidation effect with an IC_50_ value of 0.011 mg/mL. Similarly, Abdel Ghani et al. (2023) reported that the dichloromethane fraction of *S. hispanica* L. showed stronger anti-inflammatory activity with IC_50_ of 0.061 mg/mL compared to diclofenac sodium as a positive control, with an IC_50_ of 0.0179 mg/mL. This activity is attributed to the presence of diterpenes and phenolics in the dichloromethane fraction. Sterols, such as β-sitosterol **[22]**, betulinic acid **[265]**, oleanolic acid **[186]**, and β-sitosterol-3-*O*-β-D-glucoside **[25]**, are also known to exhibit anti-inflammatory activity.

#### 3.2.3 Antimicrobial properties

Essential oils from *Salvia* species, particularly those containing thujones and eucalyptol, exhibit antibacterial and antifungal properties. EOs from leaves and flowers of *S. hydrangea* showed a significant inhibitory effect on the Gram-negative bacterial species: *Pseudomonas aeruginosa*, *Shigella dysenteriae*, and *K. pneumoniae,* with a minimum inhibitory concentration of 16–62 μg/mL (Ghavam et al., 2020). Kačániová et al. (2023) reported the strong effects of *S. sclarea* EOs compared to the standard (33 mm) for the Gram bacterial strain, *Bacillus subtilis* (12 ± 1.00 mm), and the yeast, *C. albicans* (11.33 ± 0.58 mm). Taking into account the strains of fungi, the strongest activity of the tested EOs was observed toward *Aspergillus flavus* with an inhibition zone of 10.33 ± 0.58 mm. Piątczak et al. (2021) showed that the roots of *S. cadmica* demonstrated antimicrobial activity. They noticed it against two species of *Candida* and several Gram-positive bacteria, including *Bacillus cereus* and four strains of *Staphylococcus* spp. Demirpolat (2023) reported that the EOs of *Salvia* species showed varying antimicrobial activity. Promising antibacterial results against *Escherichia coli* and *Bacillus megaterium* were observed by using *S. multicaulis*. Furthermore, the EOs of *S. verbenaca* and *S. ceratophylla* were active against *K. pneumoniae* and *Staphylococcus aureus,* respectively. The active *Salvia* species against *Candida albicans* and *Candida glabrata* were *S. verbenaca* and *S. multicaulis*, with an inhibition zone of 25–28 mm, respectively.

Balaei-Kahnamoei et al. (2021) reported in their study that *S. aureus* and *E. coli* were inhibited by the chloroform fraction of aerial parts of *S. macrosiphon.* The MIC was 0.6 mg/mL for both strains. Sargazifar et al. (2024) studied that aegyptinone A **[82]**, present in the root extract of *S. santolinifolia* showed moderate antibacterial activity against *S. aureus*, *Staphylococcus epidermis*, and *B. subtilis* with a MIC of 25 μg/mL. Karadeniz-Pekgöz et al. (2024) reported the anti-bacterial activity of a 10 mg/mL concentration of chia seed methanol extract. They found efficiency against strains of *S. aureus*, *S. enterica,* and *L. monocytogenes*. Bilginoğlu et al. (2025) observed the inhibition zones (15–20 mm) presenting the antimicrobial activity of the ethanol extract of *S. aethiopis* at a concentration of 4 mg/mL. Promising results were observed against these bacterial strains: *S. epidermidis*, *Micrococcus luteus*, *B*.* cereus*, *Listeria monocytogenes*, *K. pneumoniae*, *S. dysenteriae*, and *C. albicans.* Akrimi et al. (2025) analyzed that the hexane extract of *S. officinalis* demonstrated the greatest antibacterial effect against *S. epidermidis* and *S. aureus* (MIC = 0.156 mg/mL), and the authors reported that it is due to the presence of rosmarinic acid **[1]** as the main component.

#### 3.2.4 Anticancer potential

**FIGURE 5:**
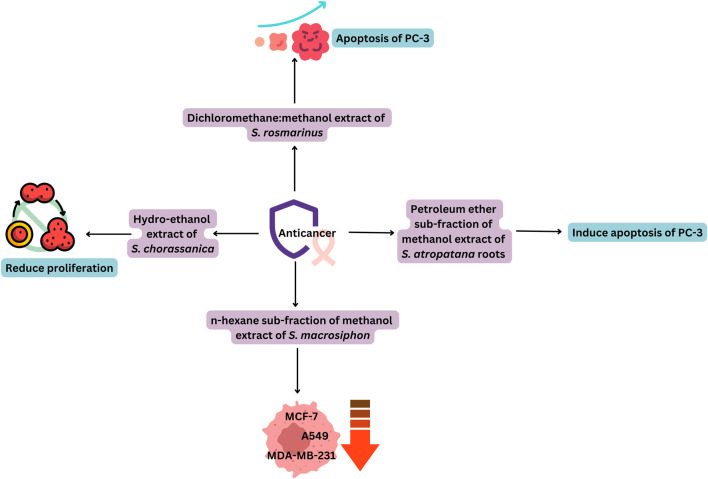
Promising anticancer activity of *Salvia* extracts.

Extracts of some *Salvia* spp. or their phytochemicals have shown the potential to inhibit carcinogenesis, proliferation, and metastasis of cancer cells (Table 4), while causing minimal damage to normal cells. The anticancer activity of the extracts was quite effective, since it showed results similar to reference anticancer drugs (Figure 5) (Ezema et al., 2022). Deng et al. (2020) identified different tanshinones in *S. miltiorrhiza*, including dihydrotanshinone I **[76]**, cryptotanshinone **[183]**, tanshinone I **[182]**, and tanshinone IIA **[232]**. These compounds exhibit a variety of pharmacological activities, including antitumor, anti-inflammatory, and antibacterial effects. A similar study by Zhao et al. (2022) showed that *S. miltiorrhiza* is enriched with liposoluble tanshinones (dihydrotanshinone I **[76]**, tanshinone I **[182]**, tanshinone IIA **[232]**, and cryptotanshinone **[183]**) and water-soluble phenolic acids (salvianolic acid A **[167]**, salvianolic acid B **[168]**, salvianolic acid C **[146]**, and rosmarinic acid **[1]**). These compounds target breast cancer cells by altering the mechanisms such as: induction of apoptosis, autophagy, and cell cycle arrest, anti-metastasis, formation of cancer stem cells, and potentiation of antitumor immunity.

**TABLE 4:** Cellular targets of anticancer phytochemicals from *Salvia* species.

Plant species	Isolated phytochemical	Cancer cells	References
*S. tebesana* Bunge	Aegyptinone A **[82]**, tebesinone B **[266]**	Michigan Cancer Foundation-7 (MCF-7), melanoma, human prostate (PC-3), and colon (C26) carcinoma	Ezema et al. (2022)
*S. lachnocalyx* Hedge	Ferruginol **[75]**, taxodione **[216]**, sahandinone **[267]**, 4-dehydrosalvilimbinol **[268]**, and labda-7,14-dien-13-ol **[269]**	Acute lymphoblastic leukemia, colorectal adenocarcinoma, and MCF-7	Mirzaei et al. (2017)
*S. lachnocalyx* Hedge	15-deoxyfuerstione **[270]**, horminone **[217]**, microstegiol **[271]**, and 14-deoxycoleon U **[272]**	Human Erythroleukemia (K562) and MCF-7	Mirzaei et al. (2020)
*S. hispanica* L	Chrysin **[273]**	Ovarian Clear Cell Carcinoma (ES2) and Ovarian Papillary Serous Adenocarcinoma (OV90)	Lim et al. (2018), Lima et al. (2020), Çelik et al. (2024)

Piątczak et al. (2021) reported that the 10 mg/mL concentration of root extract of *S. cadmica* showed a 70% reduction in cell viability against mouse L929 fibroblasts in the MTT assay (Righi et al., 2021). showed the potential cytotoxic effect of the hydromethanolic extract of *S. verbenaca* against *Artemia salina* larvae with an LC_50_ value of 0.030 mg/mL. Abdel Ghani et al. (2023) studied that the dichloromethane fraction of aerial parts of *S. hispanica* revealed moderate cytotoxic activity against the human lung cancer cell line (A-549), human prostate carcinoma (PC-3), and colon carcinoma (HCT-116) with IC_50_ values of 0.035 ± 2.1, 0.042 ± 2.3, and 0.047 ± 1.3 mg/mL, respectively.

The *n*-hexane fraction of *S. macrosiphon* was found to be potent for the lung cancer cell line (A-549), two human breast cancer cell lines (MCF-7 and MDA-MB-231), and normal cells (Human Dermal Fibroblast). The tested sample exhibited cytotoxicity with IC_50_ values of 0.02, 0.01, 0.02, and 0.02 mg/mL, respectively. A compound (3-epi manoyl oxide **[35]**) of *S. macrosiphon* was found to be potent against MCF-7 with an IC_50_ value of 15.79 ± 0.35 µM, and it showed stronger activity than etoposide (37.51 ± 0.66 µM). It also showed less toxicity toward HDF, confirming that it could be considered a potent candidate in the research and development of anticancer drugs (Balaei-Kahnamoei et al., 2021). The ethyl acetate extract of chia seeds showed a potent cytotoxic effect against liver cancer cell lines (HepG2) and pancreatic cancer (MIA PaCa-2) in humans, with an IC_50_ of 0.011 mg/mL and 0.0877 mg/mL, and a percentage of cytotoxicity at 0.01 mg/mL of 98% and 56.2%, respectively. According to considerations from the United States National Cancer Institute (NCI), a crude extract is assumed to be a promising anticancer agent if its IC_50_ value ranges between 0.03 and 0.04 mg/mL. Based on these observations, it can be assumed that all *Salvia* extracts reported could be a promising source for the development of an anticancer drug (Mohamed et al., 2024).

The *S. officinalis* propylene glycol extract revealed oncostatic properties *in vitro* and *in vivo* due to the presence of phenolics (rosmarinic acid **[1]**, protocatechuic acid **[118]**, and salicylic acid **[105]**) and triterpenoids (ursolic acid **[78]** and oleanolic acid **[24]**) (Kubatka et al., 2024). The diterpenoids present in the roots of *S. leriifolia* were purified and identified by HPLC and NMR. Then, the effect of these compounds on the cell viability of different cell lines: MIA PaCa-2, Human Gastric Cancer Cell Line (AGS), MCF-7, Human Immortalized Keratinocytes (HaCaT), and cervical cancer cells (HeLa), was evaluated by the MTT method. The diterpene pisiferal has high cytotoxicity against all investigated cell lines at a concentration between 9.3 ± 0.6 and 14.38 ± 1.4 μM (Sarhadi et al., 2022). The dichloromethane extract from *S. compressa* shoots showed moderate activity against MCF-7 and reduced cell viability to 68.2% ± 13.1% at a concentration of 0.05 mg/mL (Noorbakhsh et al., 2022). The viability of non-small cell lung cancer cells was significantly affected when treated with the ethanol extract of *S. aethiopis* at a concentration of 0.02 mg/mL, and cell viability was observed as 6.40% and 8.52% after 24 and 48 h of treatment. The IC_50_ values were reported as 0.08 and 0.05 mg/mL, respectively (Bilginoğlu et al., 2025).

#### 3.2.5 Neuroprotective effects

Different disorders of the central nervous system affect 22% of the human population worldwide. To cope with depression, drugs are used that work to modify one or more monoamine neurotransmitter systems. Different *Salvia* species are used to enhance memory, as a sedative, and for the treatment of headaches (Abdelhalim and Hanrahan, 2021). The enzymes acetylcholinesterase (AChE) and butyrylcholinesterase (BChE) are considered to be primary cholinesterase regulators. Inhibition of cholinesterase (ChE) is the most effective treatment approach for Alzheimer’s disease (AD) to date. In a normal brain, AChE represents 80% of the activity, while BuChE represents the remaining 20%. AD is characterized by an increase in the level of BuChE, while AChE activity remains unchanged or declines. Selective inhibition of BuChE is a strategy to improve memory in elderly rats (Villalta et al., 2021). Sefah et al. (2025) studied the hydroethanolic leaf extract of *S. officinalis* and found that it improves memory and alleviates lipopolysaccharide-induced neuroinflammation in mice.

**FIGURE 6:**
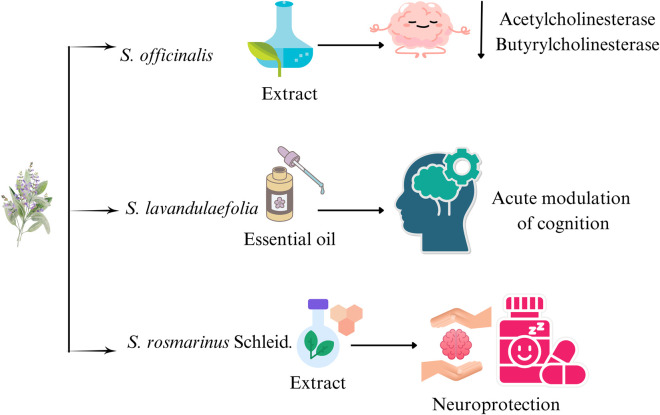
Neuroprotective effects of some *Salvia* species.

Tundis et al. (2020) reported significant activity of *S. officinalis* EO against AChE. Samples confirmed a slight difference in activity based on locality, as the IC_50_ values of 0.0476 and 0.0583 mg/mL were shown by samples from Orsomarso and Civita regions, respectively. Ertas et al. (2023) enlisted the neuroprotective effect of different *Salvia* species (Figure 6). The study reported that capsules containing 0.05 mL of *S. lavandulaefolia* EOs resulted in increased performance of secondary memory and attention tasks. A single dose of *S. lavandulaefolia* essential oil has been found to improve memory and attention task performance, increase alertness, and reduce mental fatigue during the long-term performance of difficult tasks. Similarly, the ethanolic extract (70%) of dried leaves of *S. officinalis* demonstrated significant improvements in the memory scores. Gharehbagh et al. (2023) studied that the EOs of aerial parts of *S. mirzayanii* were a potent inhibitor of AChE and BChE at a concentration of 0.05 mg/mL, with an inhibition of 72.68% and 40.6%, respectively. Ivanova et al. (2024) collected data on the neuroprotective effect of *S. verticillata*. They concluded that *S. verticillata* could be used as an adjunctive therapy in neurodegenerative diseases, including Alzheimer’s disease, due to the presence of monoterpenes, phenolic diterpenes, quercetin **[67]**, and rosmarinic acid **[1]**.

#### 3.2.6 Anti-diabetic activity

The EOs distilled from *S. officinalis* in the full flowering stage showed antidiabetic activity. IC_50_ values were 0.069, 0.022, and 0.037 mg/mL against α-amylase, α-glycosidase, and lipase, respectively (Assaggaf et al., 2022). The *in vitro* inhibitory effects of *S. officinalis* (EOs) on *α*-amylase and *α*-glucosidase were significant, with IC_50_ values of 0.081 and 0.011 mg/mL (Al-Mijalli et al., 2022). *Salvia hispanica* shows a reduction in insulin resistance that is attributed to the omega-3 content (35.64% of total fatty acids). Similarly, the dichloromethane fraction inhibited the α-amylase enzyme with an IC_50_ of 0.067 mg/mL (Abdel Ghani et al., 2023). *Salvia spinosa* was found to be a potent inhibitor (90.5% inhibition at 0.05 mg/mL) of α-glucosidase (Gharehbagh et al., 2023). Shojaeifard et al. (2023) analyzed α-glucosidase inhibitory activity of 80% MeOH extract from different *Salvia* species. At a concentration of 0.01 mg/mL, S*. santolinifolia* (94.35%), *S. multicaulis* (94.27%), and *S. eremophila* (94.02%) showed significant inhibitory activity compared to acarbose (87.88%). However, *S. nemorosa* showed 70% activity, while *S. verticillata* did not show significant activity. Several compounds: rosmarinic acid **[1]**, carnosic acid **[89]**, carnosol **[88]**, luteolin **[5]**, apigenin **[6]**, and hispidulin **[106]** are believed to be the modulators of the proposed activity.

#### 3.2.7 Other activities

Abdel Ghani et al. (2023) analyzed the anti-obesity activity of *S. hispanica* using a pancreatic lipase inhibitory assay. The dichloromethane fraction has moderate activity with an IC_50_ of 0.059 mg/mL compared to orlist (IC_50_ of 0.023 mg/mL). Vestuto et al. (2024) performed LC-MS analyses on the extracellular vesicles of hairy roots of *S. sclarea* and *S. dominica*. It highlighted the presence of ursolic acid **[78]** and oleanolic acid **[24]** derivatives. These compounds have already shown antibacterial, antioxidant, anti-inflammatory, antineoplastic, and anti-aggregant properties, along with neuroprotective effects. Kong et al. (2023) presented different mechanisms by which *S. miltiorrhiza* effectively attenuates the symptoms of Placenta-mediated pregnancy complications. In particular, *S. miltiorrhiza* and its active compounds have been shown to treat preeclampsia, mitigate the severity of fetal growth restriction, and improve adverse symptoms of spontaneous recurrent abortion. Jedidi et al. (2023) reported that the aqueous extract of *S. officinalis* flowers has protective effects against hepatorenal toxicities.

The administration of ethanol extract of aerial parts of *S. chudaei* effectively reversed the adverse effects of triton-induced hyperlipidemia. It significantly lowers cholesterol, triacylglycerol, and LDL levels. A positive effect was observed as the HDL cholesterol improved. It also enhanced antioxidant defenses and reduced markers of oxidative stress, demonstrating its protective role against metabolic dysfunction. HPLC confirmed the presence of bioactive compounds in the extract that contribute to these benefits (Tlili et al., 2025). Park et al. (2025) reported the potential to reduce inflammation of the ethanol extract of *S. miltiorrhiza*. Inflammation in RAW 264.7 macrophages was reduced by 0.08 mg/mL of ethanol extract of *S. miltiorrhiza*.

Akrimi et al. (2025) assessed the inhibitory effect of *S. officinalis* hexane extract on methicillin-resistant *S. aureus*. The concentration of 0.156 mg/mL caused a 70% inhibition of the biofilm. Therefore, the extract can improve food safety by interrupting the bacterial biofilm production process. Tsakni et al. (2025) analyzed the antiviral activity of *S. rosmarinus* leaf extract in HuhD-2 cell cultures infected with dengue virus and discovered that it has the potential for the development of antiviral drugs. Alves-Silva et al. (2023) reported the wound healing power of *S. aurea* by promoting cell migration without affecting cell viability. Huang et al. (2024) showed that *S. miltiorrhiza* is listed as a “top-tier” herb (a TCM that does not have observable toxicity) in the Sheng Nong’s Herbal Classic.

## 4 Conclusion

The potential anti-inflammatory, antibacterial, antioxidant, and neuroprotective abilities of *Salvia* species are well-studied and confirmed. It is ensured by the presence of bioactive compounds. As some species are quite popular and well-studied for their chemical profile and bioactivities, a few are least studied, leaving room for further evaluation. The present review provided recent studies on the 59 *Salvia* species reported during 2020–2025. It is observed that the variability in the chemical composition of *Salvia* can lead to significant variation in its bioactivity. There are some other factors affecting the chemical composition, such as: extraction method, plant part, solvent, and freshness of the sample. The high content of flavonoids, terpenoids, and phenolic compounds in the *Salvia* species is promising. It indicates the prospects for further study of these species for the pharmaceutical industry.

## 5 Future perspectives

Deep characterization of *Salvia* compounds along with the analysis of their ADMIT properties. Molecular Docking studies will help elucidate the molecular interaction of the compound with the receptor cell. Therefore, we can predict the binding properties and their effectiveness in curing the disease and the mechanism by which it copes. The scope of studies can be improved and optimized by considering multiple and advanced methods to ensure accuracy. It will be helpful in improving the wellness of healthcare, cosmetics, and other industries associated with natural products.

## Glossary

EOs: Essential Oils
WFO: World Flora Online
UPLC-DAD: Ultra Performance Liquid Chromatography-Diode Array Detector
ESI-MS/MS: Electrospray Ionization Tandem Mass Spectrometry
HPLC-DAD: High Performance Liquid Chromatography-Diode Array Detector
GC-MS: Gas Chromatography- Mass Spectrometry
LC-Q-Orbitrap: Liquid Chromatography coupled with a Quadrupole-Orbitrap
HRMS: High Resolution Mass Spectrometry
GLC-MS: Gas Liquid Chromatography- Mass Spectrometry
CC: Column Chromatography
TLC: Thin Layer Chromatography
NMR: Nuclear Magnetic Resonance
GC-FID: Gas Chromatography- Flame Ionization Detector
FTIR: Fourier Transform Infrared Spectroscopy
HRESIMS: High-Resolution Electrospray Ionization Mass Spectrometry
1D and 2D: One-Dimensional and Two-Dimensional
UPLC-qToF-MS: Ultra-Performance Liquid Chromatography coupled with Quadrupole Time-of-Flight Mass Spectrometry
LC-MS/MS: Liquid Chromatography- Tandem Mass Spectrometry
LC-ESI-MS: Liquid Chromatography- Electrospray Ionization Mass Spectrometry
HS-SPME: Headspace Solid-Phase Microextraction
VOCs: Volatile Organic Constituents
TMS: Tetramethylsilane
RE: Rutin Equivalent
GAE: Gallic Acid Equivalent
TPC: Total Phenolic Content
TFC: Total Flavonoid Content
DW: Dry Weight
mg/g: milligrams per Gram
mg/mL: milligrams per milliliter
COSY: Correlated Spectroscopy
NOESY: Nuclear Overhauser Effect Spectroscopy
ECD: Electron Capture Detector
EFAs: Essential Fatty Acids
DPPH: 2,2-Diphenyl-1-picrylhydrazyl
FRAP: Ferric Reducing Antioxidant Power
ABTS: 2,2′-azino-bis(3-ethylbenzothiazoline-6-sulfonic acid)
**IC**_**50**_: Half-maximal Inhibitory Concentration
**EC**_**50**_: Half Maximal Effective Concentration
**LC**_**50**_: Median Lethal Concentration
TE: Trolox Equivalent
QE: Quercetin Equivalent
MIC: Minimum Inhibitory Concentration
mg/kg: Milligram per kilogram
µM: Micromolar
MCF-7: Michigan Cancer Foundation-7
PC3: Human Prostate Carcinoma Cells
C26: Colon Carcinoma Cells
K562: Human Erythroleukemia Cells
ES2: Ovarian Clear Cell Carcinoma Cells
OV90: Ovarian Papillary Serous Adenocarcinoma Cells
MTT: 3-(4,5-dimethylthiazol-2-yl)-2,5-diphenyltetrazolium bromide
L929: Mouse Fibroblast Cell Line
MDA-MB-231: MD Anderson-Metastatic Breast-231
HDF: Human Dermal Fibroblast
HepG2: Human Liver Hepatoblastoma
MIA PaCa-2: Human Pancreatic Cancer Cell Line
AGS: Human Gastric Cancer Cell Line
HaCaT: Human Immortalized Keratinocytes
HeLa: Cervical Cancer Cells
AD: Alzheimer’s disease
AChE and BChE: Acetylcholinesterase and Butyrylcholinesterase
ChE: Cholinesterase
LDL and HDL: Low-Density Lipoprotein and High-Density Lipoprotein
TCM: Traditional Chinese Medicine
ADMET: Absorption, Distribution, Metabolism, Excretion, and Toxicity
UHPLC-HRMS: Ultra-High-Performance Liquid Chromatography–High-Resolution Mass Spectrometry

## References

[B1] Abd Rashed A. Rathi D.-N. G. (2021). Bioactive components of *salvia* and their potential antidiabetic properties: a review. Molecules 26, 3042. 10.3390/molecules26103042 34065175 PMC8161164

[B2] Abdel Ghani A. E. Al-Saleem M. S. Abdel-Mageed W. M. AbouZeid E. M. Mahmoud M. Y. Abdallah R. H. (2023). UPLC-ESI-MS/MS profiling and cytotoxic, antioxidant, anti-inflammatory, antidiabetic, and antiobesity activities of the non-polar fractions of *Salvia hispanica* L. aerial parts. Plants 12, 1062. 10.3390/plants12051062 36903922 PMC10005563

[B3] Abdelhalim A. Hanrahan J. (2021). Biologically active compounds from lamiaceae family: central nervous system effects. Stud. Nat. Prod. Chem. 68, 255–315. 10.1016/B978-0-12-819485-0.00017-7

[B4] Afonso A. F. Pereira O. R. Cardoso S. M. (2021). *Salvia* species as nutraceuticals: focus on antioxidant, antidiabetic and anti-obesity properties. Appl. Sci. 11, 9365. 10.3390/app11209365

[B5] Aissaoui M. Chalard P. Figuérédo G. Marchioni E. Zao MintJe Z. M. Benayache F. (2014). Chemical composition of the essential oil of Salvia verbenaca (L.) briq. ssp. Pseudo-jaminiana. Available online at: https://www.cabidigitallibrary.org/doi/full/10.5555/20143419107 (Accessed November 2, 2025).

[B6] Akacha B. B. Kačániová M. Mekinić I. G. Kukula-Koch W. Koch W. Orhan I. E. (2024). Sage (*Salvia officinalis* L.): a botanical marvel with versatile pharmacological properties and sustainable applications in functional foods. South Afr. J. Bot. 169, 361–382. 10.1016/j.sajb.2024.04.044

[B7] Akrimi H. Jerbi A. Elghali F. Mnif S. Aifa S. Fki L. (2025). Investigation of the bioactive properties of Tunisian sage extracts: a detailed analysis of phytochemical composition and their antioxidant, antibacterial, and antibiofilm activities. Chem. Afr. 8, 1351–1363. 10.1007/s42250-025-01240-0

[B8] Al-Mijalli S. H. Assaggaf H. Qasem A. El-Shemi A. G. Abdallah E. M. Mrabti H. N. (2022). Antioxidant, antidiabetic, and antibacterial potentials and chemical composition of *Salvia officinalis* and *Mentha suaveolens* grown wild in Morocco. Adv. Pharmacol. Pharm. Sci. 2022, 2844880. 10.1155/2022/2844880 35755940 PMC9217590

[B9] Ali M. Muhammad A. Lin Z. He H. Zhang Y. (2024). Exploring Lamiaceae-derived bioactive compounds as nature’s arsenal for sustainable pest management. Phytochem. Rev. 24, 1989–2013. 10.1007/s11101-024-09987-z

[B10] Alves-Silva J. M. Maccioni D. Cocco E. Gonçalves M. J. Porcedda S. Piras A. (2023). Advances in the phytochemical characterisation and bioactivities of *Salvia aurea* L. Essential Oil. Plants 12, 1247. 10.3390/plants12061247 36986933 PMC10056036

[B11] Al‐Jaber H. I. Obeidat S. M. Afifi F. U. Abu Zarga M. H. (2020). Aroma profile of two populations of *Salvia verbenaca* collected from two bio‐geographical zones from Jordan. Chem. Biodivers. 17, e1900553. 10.1002/cbdv.201900553 31869516

[B12] Amtaghri S. Eddouks M. (2023). Ethnopharmacology, nutritional value, therapeutic Effects,Phytochemistry, and toxicology of *Salvia hispanica* L.: a review. Curr. Top. Med. Chem. 23, 2621–2639. 10.2174/0115680266248117230922095003 37855294

[B13] Anbessa B. Lulekal E. Hymete A. Debella A. Debebe E. Abebe A. (2024). Ethnomedicine, antibacterial activity, antioxidant potential and phytochemical screening of selected medicinal plants in Dibatie district, Metekel zone, western Ethiopia. BMC Complement. Med. Ther. 24, 199. 10.1186/s12906-024-04499-x 38773522 PMC11110246

[B14] Asgarpanah J. (2021). A review on the essential oil chemical profile of *Salvia* genus from Iran. Nat. Volatiles Essent. Oils 8, 1–28. 10.37929/nveo.852794

[B15] Ashish A. Das S. Mazumder A. (2022). Ethnopharmacological importance of *Salvia hispanica* L.: an herbal panacia. Int. J. Health Sci. 6, 5949–5963. 10.53730/ijhs.v6ns5.10007

[B16] Ashraf M. V. Khan S. Misri S. Gaira K. S. Rawat S. Rawat B. (2024). High-altitude medicinal plants as promising source of phytochemical antioxidants to combat lifestyle-associated oxidative stress-induced disorders. Pharmaceuticals 17, 975. 10.3390/ph17080975 39204080 PMC11357401

[B17] Askari S. F. Avan R. Tayarani-Najaran Z. Sahebkar A. Eghbali S. (2021). Iranian *Salvia* species: a phytochemical and pharmacological update. Phytochemistry 183, 112619. 10.1016/j.phytochem.2020.112619 33373790

[B18] Assaggaf H. M. Naceiri Mrabti H. Rajab B. S. Attar A. A. Alyamani R. A. Hamed M. (2022). Chemical analysis and investigation of biological effects of *Salvia officinalis* essential oils at three phenological stages. Molecules 27, 5157. 10.3390/molecules27165157 36014393 PMC9415112

[B19] Balaei-Kahnamoei M. Eftekhari M. Ardekani M. R. S. Akbarzadeh T. Saeedi M. Jamalifar H. (2021). Phytochemical constituents and biological activities of *Salvia macrosiphon* Boiss. BMC Chem. 15, 4. 10.1186/s13065-020-00728-9 33468228 PMC7814726

[B20] Barhoumi L. M. Al-Jaber H. I. Abu Zarga M. H. (2022). A new diterpene and other constituents of *Salvia multicaulis* from Jordan. Nat. Prod. Res. 36, 4921–4928. 10.1080/14786419.2021.1912745 33899606

[B21] Belloum Z. Chalard P. Figuérédo G. Marchioni E. Zao M. Benayache F. (2014). Chemical composition of the essential oil of *Salvia verbenaca* (L.) Briq. ssp. clandestina (L.) Pugsl. Available online at: https://www.cabidigitallibrary.org/doi/full/10.5555/20143419098 (Accessed October 24, 2025).

[B22] Bilginoğlu E. Kızıl H. E. Öğütcü H. Ağar G. Bağcı Y. (2025). Pharmacological potential and bioactive components of wild anatolian sage (*Salvia aethiopis* L.). Food Sci. Nutr. 13, e70118. 10.1002/fsn3.70118 40144556 PMC11936837

[B23] Bingol Z. Kızıltaş H. Gören A. C. Kose L. P. Topal M. Durmaz L. (2021). Antidiabetic, anticholinergic and antioxidant activities of aerial parts of shaggy bindweed (*Convulvulus betonicifolia* Miller subsp.)–profiling of phenolic compounds by LC-HRMS. Heliyon 7, e06986. 10.1016/j.heliyon.2021.e06986 34027185 PMC8129935

[B24] Bjørklund G. Cruz-Martins N. Goh B. H. Mykhailenko O. Lysiuk R. Shanaida M. (2024). Medicinal plant-derived phytochemicals in detoxification. Curr. Pharm. Des. 30, 988–1015. 10.2174/1381612829666230809094242 37559241

[B25] Bojan L. Tripon M. Huiban F. Camen D. Sokolovic M. Tulcan C. (2024). Biochemical potential of *Salvia sclarea* L.: *in vitro* cultivation and chemical profiling approaches. J. Hortic. For. Biotechnol. 28, 349–354.

[B26] Brito-da-Costa A. M. Dias-da-Silva D. Gomes N. G. Dinis-Oliveira R. J. Madureira-Carvalho Á. (2021). Pharmacokinetics and pharmacodynamics of salvinorin A and *Salvia divinorum*: clinical and forensic aspects. Pharmaceuticals 14, 116. 10.3390/ph14020116 33546518 PMC7913753

[B27] Çelik Ş. Dervişoğlu G. İzol E. Sęczyk Ł. Özdemir F. A. Yilmaz M. E. (2024). Comprehensive phytochemical analysis of *Salvia hispanica* L. callus extracts using LC–MS/MS. Biomed. Chromatogr. 38, e5975. 10.1002/bmc.5975 39105236

[B28] Cristani M. Micale N. (2024). Bioactive compounds from medicinal plants as potential adjuvants in the treatment of mild *Acne vulgaris* . Molecules 29, 2394. 10.3390/molecules29102394 38792254 PMC11124055

[B29] Dabaghian F. Aalinezhad S. Delnavazi M. R. Saeedi M. (2025). *Salvia aristata* essential oil: chemical composition, cholinesterase inhibition, and neuroprotective effects against oxidative stress in PC12 cells. Res. J. Pharmacogn. 12, 65–75.

[B30] Dejene M. Dekebo A. Jemal K. Murthy H. C. A. Reddy S. G. (2025). Phytochemical screening and evaluation of anti-oxidant, anti-inflammatory and anticancer activities of leaves of *Vernonia amygdalina, Otostegia integrifolia*, and *Salvia rosmarinus* . Green Chem. Lett. Rev. 18, 2438069. 10.1080/17518253.2024.2438069

[B31] Dembińska K. Shinde A. H. Pejchalová M. Richert A. Swiontek Brzezinska M. (2025). The application of natural phenolic substances as antimicrobial agents in agriculture and food industry. Foods 14, 1893. 10.3390/foods14111893 40509420 PMC12155485

[B32] Demirpolat A. (2023). Essential oil composition analysis, antimicrobial activities, and biosystematic studies on six species of *Salvia* . Life 13, 634. 10.3390/life13030634 36983789 PMC10054517

[B33] Deng C. Shi M. Fu R. Zhang Y. Wang Q. Zhou Y. (2020). ABA-responsive transcription factor bZIP1 is involved in modulating biosynthesis of phenolic acids and tanshinones in *Salvia miltiorrhiza* . J. Exp. Bot. 71, 5948–5962. 10.1093/jxb/eraa295 32589719

[B34] Deshmukh V. P. (2022). Phytochemistry and Pharmacology of *Salvia officinalis* L.: a review. Bioact. Pharmacol. Med. Plants, 339–358. 10.1201/9781003281702-27

[B35] Ertas A. Yigitkan S. Orhan I. E. (2023). A focused review on cognitive improvement by the genus *Salvia* L.(Sage)—From ethnopharmacology to clinical evidence. Pharmaceuticals 16, 171. 10.3390/ph16020171 37259321 PMC9966473

[B36] Esmaeili G. Fatemi H. Baghani avval M. Azizi M. Arouiee H. Vaezi J. (2022). Diversity of chemical composition and morphological traits of eight Iranian wild *Salvia* species during the first step of domestication. Agronomy 12, 2455. 10.3390/agronomy12102455

[B37] Ezema C. A. Ezeorba T. P. C. Aguchem R. N. Okagu I. U. (2022). Therapeutic benefits of *Salvia* species: a focus on cancer and viral infection. Heliyon 8, e08763. 10.1016/j.heliyon.2022.e08763 35146151 PMC8819530

[B38] Ezema C. A. Aguchem R. N. Aham E. C. Ezeorba W. F. C. Okagu I. U. Ezeorba T. P. C. (2024). *Salvia africana-lutea* L.: a review of ethnobotany, phytochemistry, pharmacology applications and future prospects. Adv. Tradit. Med. 24, 703–724. 10.1007/s13596-023-00726-x

[B39] Francik S. Francik R. Sadowska U. Bystrowska B. Zawiślak A. Knapczyk A. (2020). Identification of phenolic compounds and determination of antioxidant activity in extracts and infusions of *Salvia* leaves. Materials 13, 5811. 10.3390/ma13245811 33352787 PMC7766674

[B40] Gebremeskal Y. H. Nadtochii L. A. Eremeeva N. B. Mensah E. O. Kazydub N. G. Soliman T. N. (2024). Comparative analysis of the nutritional composition, phytochemicals, and antioxidant activity of chia seeds, flax seeds, and psyllium husk. Food Biosci. 61, 104889. 10.1016/j.fbio.2024.104889

[B41] Gharehbagh H. J. Ebrahimi M. Dabaghian F. Mojtabavi S. Hariri R. Saeedi M. (2023). Chemical composition, cholinesterase, and α-glucosidase inhibitory activity of the essential oils of some Iranian native *Salvia* species. BMC Complement. Med. Ther. 23, 184. 10.1186/s12906-023-04004-w 37270541 PMC10239571

[B42] Ghavam M. Manca M. L. Manconi M. Bacchetta G. (2020). Chemical composition and antimicrobial activity of essential oils obtained from leaves and flowers of *Salvia hydrangea* DC. ex Benth. Sci. Rep. 10, 15647. 10.1038/s41598-020-73193-y 32973295 PMC7519093

[B43] Giuliani C. Giovanetti M. Lupi D. Mesiano M. P. Barilli R. Ascrizzi R. (2020). Tools to tie: flower characteristics, voc emission profile, and glandular trichomes of two mexican *Salvia* species to attract bees. Plants 9, 1645. 10.3390/plants9121645 33255733 PMC7760984

[B44] Gourich A. A. Bencheikh N. Bouhrim M. Regragui M. Rhafouri R. Drioiche A. (2022). Comparative analysis of the chemical composition and antimicrobial activity of four Moroccan north middle Atlas medicinal plants’ essential oils: rosmarinus officinalis L., Mentha pulegium L., Salvia officinalis L., and Thymus zygis subsp. gracilis (Boiss.) R. Morales. Rosmarinus Officinalis L. Morales. *Chem.* 4, 1775–1788. 10.3390/chemistry4040115

[B45] Gutiérrez-Román A. S. Gonzalez-Cortazar M. Trejo-Tapia G. Herrera-Ruiz M. Zamilpa A. Sanchéz-Mendoza E. (2022). Angiotensin-converting enzyme inhibitors from *Salvia elegans* Vahl. Nat. Prod. Res. 10.1080/14786419.2020.1758093 32347111

[B46] Hadkar V. M. Selvaraj C. I. (2023). An overview of bioactive constituents and pharmacological actions of iranian sage, 245–258.

[B47] Hafez Ghoran S. Taktaz F. Mozafari A. A. Tunçtürk M. Sekeroglu N. Kijjoa A. (2022). Uncommon terpenoids from *Salvia* species: chemistry, biosynthesis and biological activities. Molecules 27, 1128. 10.3390/molecules27031128 35164392 PMC8838292

[B48] Huang J. Zhang J. Sun C. Yang R. Sheng M. Hu J. (2024). Adjuvant role of *Salvia miltiorrhiza* bunge in cancer chemotherapy: a review of its bioactive components, health-promotion effect and mechanisms. J. Ethnopharmacol. 318, 117022. 10.1016/j.jep.2023.117022 37572929

[B49] Ihsan S. Qazi R. A. Jamila N. Bibi N. Wasil Z. Khan N. (2024). Biogenic gold nanoparticles of *Salvia* species in dyes degradation and detection of lead(II). Int. J. Environ. Sci. Technol. 21, 9637–9650. 10.1007/s13762-024-05613-9

[B50] Ivanova S. Dzhakova Z. Staynova R. Ivanov K. (2024). *Salvia verticillata* (L.)—Biological activity, chemical profile, and future perspectives. Pharmaceuticals 17, 859. 10.3390/ph17070859 39065710 PMC11280111

[B51] Jedidi S. Rtibi K. Selmi H. Aloui F. Sebai H. (2023). *Salvia officinalis* flowers extract ameliorates liver and kidney injuries induced by simultaneous intoxication with ethanol/castor oil. Physiol. Rep. 11, e15854. 10.14814/phy2.15854 37960994 PMC10643985

[B52] Jing Y. Hu J. Su Z. Cheng W. Zhang Y. Yang X. (2023). Structural characterisation and antioxidant activities *in vitro* and *in vivo* of a novel polysaccharide from *Salvia miltiorrhiza* . Nat. Prod. Res. 37, 1006–1011. 10.1080/14786419.2022.2096605 35801954

[B53] Kačániová M. Vukovic N. L. Čmiková N. Galovičová L. Schwarzová M. Šimora V. (2023). *Salvia sclarea* essential oil chemical composition and biological activities. Int. J. Mol. Sci. 24, 5179. 10.3390/ijms24065179 36982252 PMC10049179

[B54] Kalnyuk J. V. Yurkevich O. Y. Badaeva E. D. Semenov A. R. Zoshchuk S. A. Amosova A. V. (2025). Taxonomy, phylogeny, genomes, and repeatomes in the subgenera *Salvia, Sclarea*, and *Glutinaria* (*Salvia*, Lamiaceae). Int. J. Mol. Sci. 26, 6436. 10.3390/ijms26136436 40650212 PMC12249511

[B55] Kapil P. Tripathi A. K. Pal R. S. Singh S. K. Singh K. Jain D. (2025). Therapeutic potential of plant phenolic acids combating cancer drug resistance. Recent Adv. Food Nutr. Agric. 16. 10.2174/012772574X350275241230053727 39844399

[B56] Karadeniz-Pekgöz A. Turgut A. C. Çinbilgel İ. Yavuz O. (2024). Phytochemical contents and bioactivity of four endemic *Salvia* seeds from Turkey: a comparative study to chia seed. J. Food Meas. Charact. 18, 5638–5645. 10.1007/s11694-024-02594-8

[B57] Karagecili H. Yılmaz M. A. Ertürk A. Kiziltas H. Güven L. Alwasel S. H. (2023). Comprehensive metabolite profiling of *Berdav propolis* using LC-MS/MS: determination of antioxidant, anticholinergic, antiglaucoma, and antidiabetic effects. Molecules 28, 1739. 10.3390/molecules28041739 36838726 PMC9965732

[B58] Kharazian N. Dehkordi F. J. Lorigooini Z. (2024). Untargeted metabolite profiling: a comprehensive study using data analysis workflow in *Salvia* L. species (Lamiaceae). South Afr. J. Bot. 165, 101–125. 10.1016/j.sajb.2023.12.019

[B59] Khouchlaa A. Et-Touys A. Lakhdar F. Laasri F. E. El Idrissi A. E. Y. Zaakour F. (2021). Ethnomedicinal use, phytochemistry, pharmacology, and toxicology of *Salvia verbenaca* L.: a review. Biointerface Res. Appl. Chem. 12, 1437–1469. 10.33263/BRIAC122.14371469

[B60] Kızıltaş H. Bingol Z. Goren A. Alwasel S. Gülçin İ. (2023). Analysis of phenolic compounds by LC-HRMS and determination of antioxidant and enzyme inhibitory properties of *Verbascum speciousum* schrad. Rec. Nat. Prod. 17. 10.25135/rnp.370.2210.2598

[B61] Koçer M. İstifli E. S. (2022). Chemical composition and cholinesterase, tyrosinase, alpha-amylase and alpha-glucosidase inhibitory activity of the essential oil of *Salvia tomentosa* . Int. J. Plant Based Pharm. 2, 1–16.

[B62] Kong J. Li S. Li Y. Chen M. (2023). Effects of *Salvia miltiorrhiza* active compounds on placenta-mediated pregnancy complications. Front. Cell Dev. Biol. 11, 1034455. 10.3389/fcell.2023.1034455 36711034 PMC9880055

[B63] Krol A. Kokotkiewicz A. Luczkiewicz M. (2022). White sage (*Salvia apiana*)–a ritual and medicinal plant of the chaparral: plant characteristics in comparison with other *Salvia* species. Planta Med. 88, 604–627. 10.1055/a-1453-0964 33890254

[B64] Kubatka P. Mazurakova A. Koklesova L. Kuruc T. Samec M. Kajo K. (2024). *Salvia officinalis* L. exerts oncostatic effects in rodent and *in vitro* models of breast carcinoma. Front. Pharmacol. 15, 1216199. 10.3389/fphar.2024.1216199 38464730 PMC10921418

[B65] Leporini M. Bonesi M. Loizzo M. R. Passalacqua N. G. Tundis R. (2020). The essential oil of *Salvia rosmarinus* Spenn. from Italy as a source of health-promoting compounds: chemical profile and antioxidant and cholinesterase inhibitory activity. Plants 9, 798. 10.3390/plants9060798 32604753 PMC7356759

[B66] Levaya Y. Atazhanova G. Gabe V. Badekova K. (2025). A review of botany, phytochemistry, and biological activities of eight *salvia* species widespread in Kazakhstan. Molecules 30, 1142. 10.3390/molecules30051142 40076365 PMC11901606

[B67] Li B. Li J. Chai Y. Huang Y. Li L. Wang D. (2021). Targeted mutagenesis of CYP76AK2 and CYP76AK3 in *Salvia miltiorrhiza* reveals their roles in tanshinones biosynthetic pathway. Int. J. Biol. Macromol. 189, 455–463. 10.1016/j.ijbiomac.2021.08.112 34419551

[B68] Lim W. Ryu S. Bazer F. W. Kim S. Song G. (2018). Chrysin attenuates progression of ovarian cancer cells by regulating signaling cascades and mitochondrial dysfunction. J. Cell. Physiol. 233, 3129–3140. 10.1002/jcp.26150 28816359

[B69] Lima A. P. B. Almeida T. C. Barros T. M. B. Rocha L. C. M. Garcia C. C. M. da Silva G. N. (2020). Toxicogenetic and antiproliferative effects of chrysin in urinary bladder cancer cells. Mutagenesis 35, 361–371. 10.1093/mutage/geaa021 32789469

[B70] Liu W. Cui X. Zhong Y. Ma R. Liu B. Xia Y. (2023). Phenolic metabolites as therapeutic in inflammation and neoplasms: molecular pathways explaining their efficacy. Pharmacol. Res. 193, 106812. 10.1016/j.phrs.2023.106812 37271425

[B71] Luca S. V. Skalicka-Woźniak K. Mihai C.-T. Gradinaru A. C. Mandici A. Ciocarlan N. (2023). Chemical profile and bioactivity evaluation of *Salvia* species from Eastern Europe. Antioxidants 12, 1514. 10.3390/antiox12081514 37627509 PMC10451821

[B72] Maciel M. S. P. Dos Reis A. S. Fidelis Q. C. (2022). Antileishmanial potential of species from the family Lamiaceae: chemical and biological aspects of non-volatile compounds. Acta Trop. 228, 106309. 10.1016/j.actatropica.2022.106309 35032468

[B73] Maleš I. Dragović-Uzelac V. Jerković I. Zorić Z. Pedisić S. Repajić M. (2022). Non-volatile and volatile bioactives of *Salvia officinalis L., Thymus serpyllum L.* and *Laurus nobilis L.* extracts with potential use in the development of functional beverages. Antioxidants 11, 1140. 10.3390/antiox11061140 35740037 PMC9220411

[B74] Mátis A. Malkócs T. Kuhn T. Laczkó L. Moysiyenko I. Szabó A. (2023). Hiding in plain sight: integrative analyses uncover a cryptic *salvia* species in Europe. TAXON 72, 78–97. 10.1002/tax.12818

[B75] Mirzaei H. H. Firuzi O. Schneider B. Baldwin I. T. Jassbi A. R. (2017). Cytotoxic diterpenoids from the roots of *Salvia lachnocalyx* . Rev. Bras. Farmacogn. 27, 475–479. 10.1016/j.bjp.2017.01.009

[B76] Mirzaei H. H. Firuzi O. Jassbi A. R. (2020). Diterpenoids from roots of *Salvia lachnocalyx*; *in-silico* and *in-vitro* toxicity against human cancer cell lines. Iran. J. Pharm. Res. IJPR 19, 85. 10.22037/ijpr.2019.15429.13095 33841524 PMC8019878

[B77] Mohamed H. R. El-Wakil E. A. Hamed M. M. El-Shahid Z. A. E.-K. (2024). Phytochemical profiling, cytotoxicity and anti-inflammatory potential of *Salvia hispanica* L. seeds. Egypt. J. Chem. 67, 0–422. 10.21608/ejchem.2024.303803.10000

[B78] Mokhtar A. Souhila T. Nacéra B. Amina B. Alghonaim M. I. Öztürk M. (2023). *In vitro* antibacterial, antioxidant, anticholinesterase, and antidiabetic activities and chemical composition of *Salvia balansae* . Molecules 28, 7801. 10.3390/molecules28237801 38067531 PMC10708212

[B79] Moshari-Nasirkandi R. Alirezalu A. Chamanabad H. R. M. Amato J. Alipour H. Asghari A. (2024). Screening of native wild *Salvia nemorosa* populations for chemical compositions, antioxidant activity and UHPLC-HRMS profiling. Sci. Rep. 14, 32064. 10.1038/s41598-024-83756-y 39738427 PMC11685535

[B80] Motyka S. Koc K. Ekiert H. Blicharska E. Czarnek K. Szopa A. (2022). The current state of knowledge on *Salvia hispanica* and *Salviae hispanicae semen* (chia seeds). Molecules 27, 1207. 10.3390/molecules27041207 35208997 PMC8877361

[B81] Mrabti H. N. El Menyiy N. Charfi S. Saber M. Bakrim S. Alyamani R. A. (2022). Phytochemistry and biological properties of *Salvia verbenaca* L.: a comprehensive review. Biomed. Res. Int. 2022, 3787818. 10.1155/2022/3787818 35655480 PMC9155978

[B82] Mróz M. Kusznierewicz B. (2023). Phytochemical screening and biological evaluation of Greek sage (*Salvia fruticosa* Mill.) extracts. Sci. Rep. 13, 22309. 10.1038/s41598-023-49695-w 38102229 PMC10724190

[B83] Mutlu M. Bingol Z. Ozden E. Köksal E. Ertürk A. Goren A. (2025). Antioxidant, and enzyme inhibition effects of chia (*Salvia hispanica*) seed oil: a comprehensive phytochemical screening using LC-HR/MS. Electron. J. Biotechnol. 74, 41–53. 10.1016/j.ejbt.2024.12.002

[B84] Nasir A. Afridi O. K. Ullah S. Khan H. Bai Q. (2024). Mitigation of sciatica injury-induced neuropathic pain through active metabolites derived from medicinal plants. Pharmacol. Res. 200, 107076. 10.1016/j.phrs.2024.107076 38237646

[B85] Nikolova M. Aneva I. (2017). “European species of Genus *salvia*: distribution, chemodiversity and biological activity,” in Salvia biotechnology. Editors Georgiev V. Pavlov A. (Cham: Springer International Publishing), 1–30. 10.1007/978-3-319-73900-7_1

[B86] Nilofar N. Bahadırlı N. P. Elhawary E. A. Eldahshan O. Singab A. N. Saka E. (2024). Exploring the chemical composition and biological effects of four *salvia* hybrids: an innovative perspective on functional yields. eFood 5, e70012. 10.1002/efd2.70012

[B87] Niu W. Miao J. Li X. Guo Q. Deng Z. Wu L. (2022). Metabolomics combined with systematic pharmacology reveals the therapeutic effects of *Salvia miltiorrhiza* and *Radix Pueraria lobata* herb pair on type 2 diabetes rats. J. Funct. Foods 89, 104950. 10.1016/j.jff.2022.104950

[B88] Noorbakhsh F. Zare S. Firuzi O. Sakhteman A. Chandran J. N. Schneider B. (2022). Phytochemical analysis and biological activity of *Salvia compressa* Vent. Iran. J. Pharm. Res. IJPR 21, e127031. 10.5812/ijpr-127031 36942072 PMC10024313

[B89] Nwafor E.-O. Lu P. Li J.-W. Zhang Q.-Q. Qi D.-L. Liu Z.-D. (2021). Traditional Chinese medicine of *Salvia miltiorrhiza* Bunge: a review of phytochemistry, pharmacology and pharmacokinetics. Tradit. Med. Res. 6, 35. 10.53388/tmr20201027204

[B90] Nwozo O. S. Effiong E. M. Aja P. M. Awuchi C. G. (2023). Antioxidant, phytochemical, and therapeutic properties of medicinal plants: a review. Int. J. Food Prop. 26, 359–388. 10.1080/10942912.2022.2157425

[B91] Onder A. Izgi M. N. Cinar A. S. Zengin G. Yilmaz M. A. (2022). The characterization of phenolic compounds *via* LC-ESI-MS/MS, antioxidant, enzyme inhibitory activities of *Salvia absconditiflora, Salvia sclarea,* and *Salvia palaestina*: a comparative analysis. South Afr. J. Bot. 150, 313–322. 10.1016/j.sajb.2022.07.030

[B92] Ortiz-Mendoza N. Aguirre-Hernández E. Fragoso-Martínez I. González-Trujano M. E. Basurto-Peña F. A. Martínez-Gordillo M. J. (2022). A review on the ethnopharmacology and phytochemistry of the neotropical sages (*Salvia* subgenus Calosphace; Lamiaceae) emphasizing Mexican species. Front. Pharmacol. 13, 867892. 10.3389/fphar.2022.867892 35517814 PMC9061990

[B93] Pachura N. Zimmer A. Grzywna K. Figiel A. Szumny A. Łyczko J. (2022). Chemical investigation on *Salvia officinalis* L. Affected by multiple drying techniques–the comprehensive analytical approach (HS-SPME, GC–MS, LC-MS/MS, GC-O and NMR). Food Chem. 397, 133802. 10.1016/j.foodchem.2022.133802 35914462

[B94] Park S. Kim Y. Kim J. Jeon H.-J. Kim Y. G. Park W. T. (2025). Phytochemical and pharmacological profiles of various *Salvia miltiorrhiza* Bunge cultivars grown in Korea. Ind. Crops Prod. 227, 120800. 10.1016/j.indcrop.2025.120800

[B95] Pezantes-Orellana C. German Bermúdez F. Matías De la Cruz C. Montalvo J. L. Orellana-Manzano A. (2024). Essential oils: a systematic review on revolutionizing health, nutrition, and omics for optimal well-being. Front. Med. 11, 1337785. 10.3389/fmed.2024.1337785 38435393 PMC10905622

[B96] Piątczak E. Owczarek A. Lisiecki P. Gonciarz W. Kozłowska W. Szemraj M. (2021). Identification and quantification of phenolic compounds in *Salvia cadmica* Boiss. and their biological potential. Ind. Crops Prod. 160, 113113. 10.1016/j.indcrop.2020.113113

[B97] Poulios E. Giaginis C. Vasios G. K. (2020). Current State of the Art on the Antioxidant Activity of Sage (*Salvia* spp.) and its Bioactive Components. Planta Med. 86, 224–238. 10.1055/a-1087-8276 31975363

[B98] Rashwan H. M. Mohammed H. E. El-Nekeety A. A. Hamza Z. K. Abdel-Aziem S. H. Hassan N. S. (2021). Bioactive phytochemicals from *Salvia officinalis* attenuate cadmium-induced oxidative damage and genotoxicity in rats. Environ. Sci. Pollut. Res. 28, 68498–68512. 10.1007/s11356-021-15407-y 34275073

[B99] Righi N. Boumerfeg S. Deghima A. Fernandes P. A. Coelho E. Baali F. (2021). Phenolic profile, safety assessment, and anti-inflammatory activity of *Salvia verbenaca* L. J. Ethnopharmacol. 272, 113940. 10.1016/j.jep.2021.113940 33631275

[B100] Saleem A. Akhtar M. F. Sharif A. Akhtar B. Siddique R. Ashraf G. M. (2022). Anticancer, cardio-protective and anti-inflammatory potential of natural-sources-derived phenolic acids. Molecules 27, 7286. 10.3390/molecules27217286 36364110 PMC9656250

[B101] Salimikia I. Mirzania F. (2022). A review of traditional uses, phytochemistry, and pharmacology of *Salvia chloroleuca* Rech. f. and Aellen. Curr. Tradit. Med. 8, e010422202998. 10.2174/2215083808666220401152135

[B102] Samuel H. S. Undie D. Okibe G. Ochepo O. Mahmud F. Ilumunter M. (2024). Antioxidant and phytochemical classification of medicinal plants used in the treatment of cancer disease. J. Chem. Lett. 1. 10.22034/jchemlett.2024.453132.1179

[B103] Sargazifar Z. Esmaeilzadeh Kashi M. Tazik Z. Mottaghipisheh J. Hosseini S. H. Stuppner H. (2024). A new diterpenoid from *Salvia santolinifolia* boiss. Nat. Prod. Res. 38, 1570–1576. 10.1080/14786419.2022.2161538 36576048

[B104] Sarhadi E. Ebrahimi S. N. Hadjiakhoondi A. Abbas-Mohammadi M. Manayi A. Parisi V. (2022). Cytotoxic abietane diterpenoids from *Salvia leriifolia* Benth. Phytochemistry 202, 113310. 10.1016/j.phytochem.2022.113310 35863476

[B105] Sefah B. Ashie Y. Osafo N. Mante P. K. (2025). Hydroethanolic extract of *Salvia officinalis* L. leaves improves memory and alleviates neuroinflammation in ICR mice. Sci. World J. 2025, 2198542. 10.1155/tswj/2198542 40151497 PMC11949616

[B106] Shahraki Z. Taghizadeh M. S. Niazi A. Rowshan V. Moghadam A. (2024). Enhancing bioactive compound production in *Salvia mirzayanii* through elicitor application: insights from *in vitro* and *in silico* studies. Food Biosci. 60, 104185. 10.1016/j.fbio.2024.104185

[B107] Shojaeifard Z. Moheimanian N. Jassbi A. R. (2023). Comparison of inhibitory activities of 50 *salvia* species against α-Glucosidase. J. Diabetes Metab. Disord. 22, 1685–1693. 10.1007/s40200-023-01301-6 37975136 PMC10638318

[B108] Shou M. Lin Q. Xu Y. Zhu R. Shi M. Kai G. (2025). New insights of advanced biotechnological engineering strategies for tanshinone biosynthesis in *Salvia miltiorrhiza* . Plant Sci. 352, 112384. 10.1016/j.plantsci.2025.112384 39756484

[B109] Stanciu G. Lupsor S. Oancea E. Mititelu M. (2022). Biological activity of essential sage oil. J. Sci. Arts 22, 211–218. 10.46939/j.sci.arts-22.1-b02

[B110] Stanković J. S. K. Srećković N. Mišić D. Gašić U. Imbimbo P. Monti D. M. (2020). Bioactivity, biocompatibility and phytochemical assessment of lilac sage, *Salvia verticillata* L.(Lamiaceae)-A plant rich in rosmarinic acid. Ind. Crops Prod. 143, 111932. 10.1016/j.indcrop.2019.111932

[B111] Tasheva K. Sulikovska I. Georgieva A. Djeliova V. Lozanova V. Vasileva A. (2025). Phytochemical profile, antioxidant capacity and anticancer potential of water extracts from *in vitro* cultivated *Salvia aethiopis* . Molecules 30, 1427. 10.3390/molecules30071427 40286005 PMC11990555

[B112] Terzi H. Yalçın H. Yıldız M. Zengin G. Pehlivan E. Uba A. I. (2025). Exogenous nitric oxide induces production of phenolic compounds, enzyme inhibitory properties and antioxidant capacity through activating the phenylpropanoid pathway in sage (*Salvia officinalis*) leaves. South Afr. J. Bot. 180, 811–819. 10.1016/j.sajb.2025.03.044

[B113] Tlili M. L. Laib I. Hammoudi R. Hadj‐Mohamed M. Laouini S. E. Bouafia A. (2025). Therapeutic efficacy of *Salvia chudaei* ethanol extract in hyperlipidemia, hyperglycemia, and oxidative stress in Triton X‐100‐Induced Wistar rats. Chem. Biodivers. 22, e202403017. 10.1002/cbdv.202403017 39739250

[B114] Tock M. L. A. Chen W. Combrinck S. Sandasi M. Kamatou G. P. P. Viljoen A. M. (2021). Exploring the phytochemical variation of non-volatile metabolites within three South African *Salvia* species using UPLC-MS fingerprinting and chemometric analysis. Fitoterapia 152, 104940. 10.1016/j.fitote.2021.104940 34029652

[B115] Tran T. G. Nguyen V. H. Nguyen V. T. (2024). Physicochemical, phytochemical and antioxidant properties of medicinal plant roots Dan sam (*Salvia miltiorrhiza* Bunge) prepared under different drying conditions. Cogent Food Agric. 10, 2420843. 10.1080/23311932.2024.2420843

[B116] Tsakni A. Kyriakopoulou E. Letsiou S. Halvatsiotis P. Rigopoulos H. Vassilaki N. (2025). *In vitro* determination of antimicrobial, antioxidant and antiviral properties of Greek plant extracts. Microorganisms 13, 177. 10.3390/microorganisms13010177 39858945 PMC11767790

[B117] Tsitsigianni E. Tomou E.-M. Almpani C. Rallis M. C. Skaltsa H. (2023). Biological activities of Lamiaceae species: Bio-Guided isolation of active metabolites from *Salvia officinalis L* . Agronomy 13, 1224. 10.3390/agronomy13051224

[B118] Tundis R. Leporini M. Bonesi M. Rovito S. Passalacqua N. G. (2020). *Salvia officinalis L.* from Italy: a comparative chemical and biological study of its essential oil in the mediterranean context. Molecules 25, 5826. 10.3390/molecules25245826 33321838 PMC7763040

[B119] Uysal I. Koçer O. Mohammed F. S. Lekesiz Ö. Doğan M. Şabik A. E. (2023). Pharmacological and nutritional properties: Genus *Salvia* . Adv. Pharmacol. Pharm. 11, 140–155. 10.13189/app.2023.110206

[B120] Vestuto V. Conte M. Vietri M. Mensitieri F. Santoro V. Di Muro A. (2024). Multiomic profiling and neuroprotective bioactivity of *Salvia* Hairy Root-Derived extracellular vesicles in a cellular model of parkinson’s disease. Int. J. Nanomedicine 19, 9373–9393. 10.2147/IJN.S479959 39286353 PMC11403015

[B121] Villalta G. Salinas M. Calva J. Bec N. Larroque C. Vidari G. (2021). Selective BuChE inhibitory activity, chemical composition, and enantiomeric content of the essential oil from *Salvia leucantha* Cav. collected in Ecuador. Plants 10, 1169. 10.3390/plants10061169 34207496 PMC8227987

[B122] Yilmaz M. A. Ertas A. Yener I. Olmez O. T. Firat M. Temel H. (2022). Development and validation of a novel LC–MS/MS method for the quantitation of 19 fingerprint phytochemicals in *salvia* species: a chemometric approach. J. Chromatogr. Sci. 60, 770–785. 10.1093/chromsci/bmab125 34725681

[B123] Zhao H. Han B. Li X. Sun C. Zhai Y. Li M. (2022). *Salvia miltiorrhiza* in breast cancer treatment: a review of its phytochemistry, derivatives, nanoparticles, and potential mechanisms. Front. Pharmacol. 13, 872085. 10.3389/fphar.2022.872085 35600860 PMC9117704

[B124] Zheng X. Kadir A. Zheng G. Jin P. Qin D. Maiwulanjiang M. (2020). Antiproliferative abietane quinone diterpenoids from the roots of *Salvia deserta* . Bioorg. Chem. 104, 104261. 10.1016/j.bioorg.2020.104261 32920364

[B125] Zhong Z. Chen M. Zhu C. Li Y. Zhou M. Wang C. (2025). Phytochemicals from *Salvia substolonifera* with anti‐angiogenic properties and substolide H decreased oxygen‐induced retinal neovascularization. Chem. Biodivers. 22, e202401427. 10.1002/cbdv.202401427 39617721

[B126] Zhumaliyeva G. Zhussupova A. Zhusupova G. E. Błońska-Sikora E. Cerreto A. Omirbekova N. (2023). Natural compounds of *Salvia* L. genus and molecular mechanism of their biological activity. Biomedicines 11, 3151. 10.3390/biomedicines11123151 38137372 PMC10740457

